# Aqueous-Phase Reaction Mechanisms of Small α-Dicarbonyls in the Presence of Phthalate Esters

**DOI:** 10.3390/toxics13040272

**Published:** 2025-04-02

**Authors:** Wenjian Li, Qiuju Shi, Jiaxin Wang, Ruize Ma, Yanpeng Gao, Yuemeng Ji

**Affiliations:** 1 Guangdong-Hong Kong-Macao Joint Laboratory for Contaminants Exposure and Health, Guangdong Key Laboratory of Environmental Catalysis and Health Risk Control, Institute Environmental Health and Pollution Control, Guangdong University of Technology, Guangzhou 510006, China; gin668559@163.com (W.L.); shiqiuju0811@163.com (Q.S.); wzzz0704@163.com (J.W.); maruize7@163.com (R.M.); gaoyp2016@gdut.edu.cn (Y.G.); 2 Guangdong Basic Research Center of Excellence for Ecological Security and Green Development, Key Laboratory of City Cluster Environmental Safety and Green Development of the Ministry of Education, School of Environmental Science and Engineering, Guangdong University of Technology, Guangzhou 510006, China

**Keywords:** glyoxal, methylglyoxal, emerging contaminants, phthalate esters, oligomerization, aqueous phase, secondary organic aerosol

## Abstract

Oligomerization of glyoxal (GL) and methylglyoxal (MG) plays a vital role in secondary organic aerosol (SOA) formation in aqueous aerosols. However, the influence of emerging contaminants on the oligomerization of GL and MG remains unclear. Therefore, using quantum chemical and kinetic calculations, we investigated the oligomerization of GL and MG in the presence of phthalate esters (PAEs), including dimethyl phthalate (DMP), diethyl phthalate (DEP), dipropyl phthalate (DPP), and dibutyl phthalate (DBP), and the role of PAEs in the oligomerization. Our findings indicate that the direct PAE-mediated oligomerization of GL and MG is hindered due to the lack of reactive sites. However, the oligomerization of GL and MG is readily mediated by the hydrolysates of PAEs, which are the preferred forms of PAEs in weakly acidic aerosols, attributable to the additional -OH groups. The mechanisms show that the indirect PAE-mediated oligomerization proceeds via three-step reactions, including nucleophilic attack on carbenium ions, hydration, and deprotonation, which are thermodynamically and kinetically favorable. Our results reveal that the role of PAEs in the GL/MG oligomerization needs to be emphasized, particularly in conditions with a pH value approaching neutrality.

## 1. Introduction

Fine particulate matter (PM) causes atmospheric visibility reduction [1], adverse health effects [2,3], and direct and indirect effects on weather and climate patterns [4,5,6,7,8]. Secondary organic aerosol (SOA), as a major constituent of PM [9,10,11], mainly originates from the photooxidation reactions of volatile organic compounds (VOCs). Small α-dicarbonyl compounds (SαDs), as a typical class of VOCs, are critical precursors for aqueous SOA formation [12] due to the high polarity [13] and Henry’s law constants [14,15,16]. Evidence shows that the aqueous-phase chemistry of SαDs contributes to a global SOA budget of 11 Tg C a^−1^ [17]. Therefore, the aqueous reactions of SαDs are significant to SOA formation [18,19,20], particularly through the production of oligomers.

Glyoxal (GL) and methylglyoxal (MG), as typical SαDs, greatly originate from the gas-phase oxidation of biogenic isoprene and anthropogenic aromatics [21,22,23,24]. The global sources of GL and MG are estimated to be 45 and 140 Tg yr^−1^, respectively [17]. The aqueous-phase oligomerization of GL and MG has been identified as an important source of SOA formation, accounting for 53% of the SOA formation in the Pearl River Delta region [25] and 26% in the Beijing-Tianjin-Hebei region [26]. Previous studies have pointed out that oligomers are formed through acid-catalyzed reactions of GL and MG in weakly acidic aerosols [27,28]. However, their aqueous-phase oligomerization is not only complex but also significantly influenced by coexisting species in multicomponent aerosol particles. For example, aldol condensation of carbonyl compounds in tropospheric aerosols is accelerated by catalysis of inorganic salts (e.g., ammonium ions and carbonate ions) [29]. Moreover, the high-molecular-weight oligomers are generated by the aqueous-phase reactions of GL in the presence of urea and cyanamide [30,31]. In contrast, the oligomerization of GL and MG with ammonia and organic amines forms nitrogen-containing chromophores [32,33] but hinders SOA formation [34]. Therefore, the complex roles that coexisting species play in GL/MG oligomerization need to be further clarified. Phthalate esters (PAEs), a class of emerging contaminants, are extensively used as plasticizers in plastics [35] and have been widely detected in atmospheric particles [36,37]. The concentration of PAEs in the particle phase is up to the ppb level during polluted periods in megacities [38]. Hence, it is necessary to investigate the oligomerization of GL and MG in the presence of PAEs and the influence of PAEs on SOA formation.

In this study, the aqueous-phase chemistry of GL and MG in the presence of typical PAEs was investigated to simulate the oligomerization of α-dicarbonyl in the presence of PAEs in weakly acidic atmospheric cloud/fog droplets, using quantum chemistry calculations. Dimethyl phthalate (DMP), diethyl phthalate (DEP), dipropyl phthalate (DPP), and dibutyl phthalate (DBP) were selected as the representatives of PAEs due to their widespread use as plasticizers and relatively high concentrations in PM [39]. The mechanisms of PAE-mediated oligomerization of GL and MG were established and compared. Kinetics data, including rate constants and product distribution, were estimated and characterized by the conventional transition state theory. Additionally, the implication of emerging contaminants to SOA formation through GL/MG oligomerization was also discussed.

## 2. Methods

All density functional theory (DFT) calculations in the present work were performed using the Gaussian 09 program package [40]. Geometries of stationary points (SPs), including reactants, transition states (TSs), intermediates, and products, were optimized at the M06-2X functional [41] with the 6-311G(d,p) basis set [42], i.e., at the M06-2X/6-311G(d,p) level. This computational approach has demonstrated excellent performance in modeling the oligomerization of α-dicarbonyl in the aqueous phase, as evidenced by previous studies [34,43,44]. The consistency of calculated growth rates of SOA in the theoretical study (1.41 µg m^−3^ h^−1^) with those values of the experimental data (1.44 µg m^−3^ h^−1^) [34] indicates the reliability of theoretical calculations. It implies that the M06-2X method provides a reliable description of the kinetics of the α-dicarbonyl oligomerization. The solvent effects of water were simulated using the solvation model based on density (SMD) [45]. Harmonic vibrational frequency analyses were carried out at the same level as geometry optimization to confirm whether the SP is a TS (with only one imaginary frequency) or a minimum (without imaginary frequencies). Intrinsic reaction coordinate (IRC) calculations were carried out to verify that each TS connected the corresponding reactants and products. Barrierless processes, characterized by the absence of TSs, were verified by scanning the pointwise potential curve (PPC) [46]. Single-point energy (SPE) calculations were performed at the M06-2X/6-311 + G(3df,3pd) level on the optimized geometries to improve the accuracy of potential energy surfaces (PESs). The dual-level approach (denoted as M06-2X//M06-2X) combines SPEs calculated with M06-2X/6-311 + G(3df,3pd) and geometries optimized at M06-2X/6-311G(d,p). The natural bond orbital (NBO) method was employed to analyze the natural charges of key species [47].

Rate constants for reactions involving well-defined transition states (TSs) were determined using conventional transition state theory (CTST) [48,49,50] based on the refined PESs. To account for realistic aqueous-phase conditions, bimolecular reaction kinetics were calculated by incorporating solvent cage effects [51], free volume constraints, and diffusion-limited interactions [51,52,53]. Barrierless processes are dominated by the diffusion-limited effects, with their rate constants directly assigned as diffusion-limited rates. Detailed computational methods for kinetics are provided in Section S1.1 within the Supplementary Material.

## 3. Results and Discussion

### 3.1. Initial Hydrolysis Pathways of PAEs

**Figure 1:**
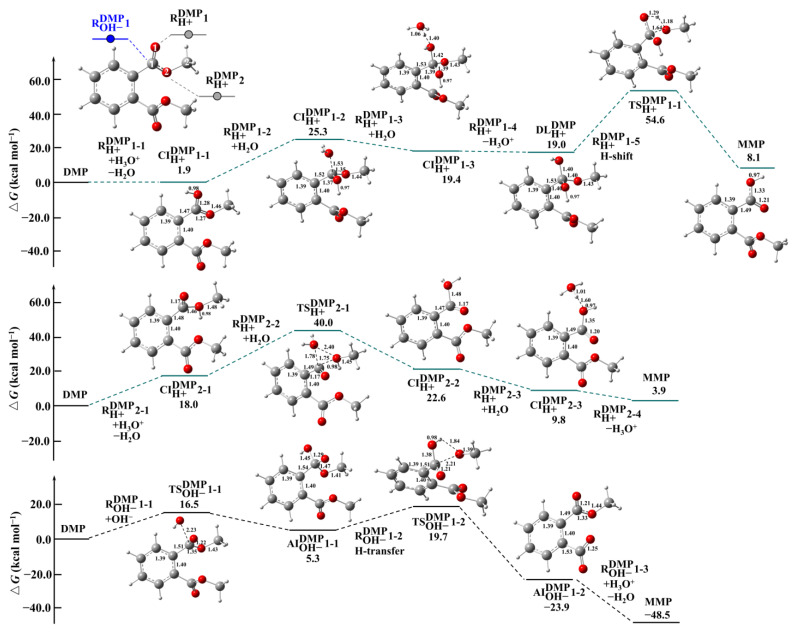
The PESs for the indirect hydrolysis reactions of DMP were obtained at the level of M06-2X//M06-2X. The numbers denote charge values (in e). 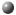 C; 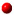 O; 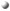 H.

The calculated PESs for the possible hydrolysis reaction pathways of PAEs are shown in Figure 1, Figures S3 and S4. The initial hydrolysis reactions of four PAEs occur via two distinct pathways: direct hydrolysis ($R_{H2O}^{X}$) and indirect hydrolysis mediated by H^+^ ($R_{H+}^{X}$) or OH^−^ ($R_{\mathrm{OH}-}^{X}$). The nomenclature $R_{Y}^{X}$ is established for each pathway for the convenience of the following discussion, where X denotes the four target PAEs (DMP, DEP, DPP, and PBP), and Y represents an elementary reaction type (direct hydrolysis and indirect hydrolysis mediated by OH^−^ or H^+^). The first and second digits in the nomenclature denote the reaction site and elementary reaction step, respectively. The optimized geometries of the four PAEs and the NPA charge values of key species are shown in Figures S1 and S2. The optimized structures of key species are presented in Figures S5–S8.

As shown in Figure S3, the direct hydrolysis pathways of four PAEs ($R_{H2O}^{X}1$) possess large Δ*G*^‡^ values with the range of 58.7–60.1 kcal mol^−1^, implying that these pathways are thermodynamically and kinetically unfavorable. Therefore, the indirect hydrolysis reactions of PAEs mediated by H^+^ and OH^−^ are mainly discussed in the following study.

For DMP, the indirect hydrolysis of DMP mediated by H^+^ starts with barrierless protonation at the O1 ($R_{H+}^{\mathrm{DMP}}1$) and O2 atoms ($R_{H+}^{\mathrm{DMP}}2$) of -COOCH_3_ group, with the Δ*G*_r_ values of 1.9 and 18.0 kcal mol^−1^, respectively, forming cationic intermediates (CIs), ${\mathrm{CI}}_{H+}^{\mathrm{DMP}}1-1$ and ${\mathrm{CI}}_{H+}^{\mathrm{DMP}}2-1$. Subsequently, H_2_O addition to ${\mathrm{CI}}_{H+}^{\mathrm{DMP}}2-1$ ($R_{H+}^{\mathrm{DMP}}2-2$) proceeds via a transition state (TS) with the Δ*G*^‡^ value of 22.0 kcal mol^−1^ to form ${\mathrm{CI}}_{H+}^{\mathrm{DMP}}2-2$, while H_2_O addition to ${\mathrm{CI}}_{H+}^{\mathrm{DMP}}1-1$ ($R_{H+}^{\mathrm{DMP}}1-2$) is barrierless and endothermic with the Δ*G*_r_ of 23.4 kcal mol^−1^ to yield ${\mathrm{CI}}_{H+}^{\mathrm{DMP}}1-2$. It indicates the more favorable formation of ${\mathrm{CI}}_{H+}^{\mathrm{DMP}}1-2$ than ${\mathrm{CI}}_{H+}^{\mathrm{DMP}}2-2$. The subsequent reaction of ${\mathrm{CI}}_{H+}^{\mathrm{DMP}}1-2$ involves hydration ($R_{H+}^{\mathrm{DMP}}1-3$), deprotonation ($R_{H+}^{\mathrm{DMP}}1-4$), and intramolecular proton transfer ($R_{H+}^{\mathrm{DMP}}1-5$). $R_{H+}^{\mathrm{DMP}}1-3$ and $R_{H+}^{\mathrm{DMP}}1-4$ are barrierless, while $R_{H+}^{\mathrm{DMP}}1-5$ possesses a high activation barrier with the Δ*G*^‡^ value of 35.6 kcal mol^−1^, implying that it is the rate-limiting step in the H^+^-mediated hydrolysis reactions of DMP.

On the other hand, the nucleophilic attack of DMP by OH^−^ occurs at the C1 atom of -COOCH_3_ group ($R_{\mathrm{OH}-}^{\mathrm{DMP}}1-1$), with the Δ*G*^‡^ value of 16.5 kcal mol^−1^, forming an anionic intermediate (AI), ${\mathrm{AI}}_{\mathrm{OH}-}^{\mathrm{DMP}}1-1$. ${\mathrm{AI}}_{\mathrm{OH}-}^{\mathrm{DMP}}1-1$ then undergoes the intramolecular proton transfer ($R_{\mathrm{OH}-}^{\mathrm{DMP}}1-2$) to form ${\mathrm{AI}}_{\mathrm{OH}-}^{\mathrm{DMP}}\;1-2$ and methanol (CH_3_OH), with the Δ*G*^‡^ value of 14.4 kcal mol^−1^. The neutralization of ${\mathrm{AI}}_{\mathrm{OH}-}^{\mathrm{DMP}}1-2$ is barrierless and strongly exothermic, with the Δ*G*_r_ value of −24.6 kcal mol^−1^, to form monomethyl phthalate (MMP). Hence, compared with the H^+^-mediated hydrolysis reaction, the OH^−^-mediated hydrolysis reaction of DMP is more kinetically feasible.

Similarly, for the other three PAEs, the H^+^-mediated hydrolysis reactions also proceed via protonation, hydration, and deprotonation, and the OH^−^-mediated hydrolysis reactions proceed via OH^−^-addition, intramolecular proton transfer and neutralization, forming phthalate monoesters. To identify the most preferred hydrolysis reaction, the rate constants (*k*) and the half-lives (t_1/2_) of H^+^/OH^−^-mediated hydrolysis reactions were calculated and are listed in Tables S1 and S2. For DMP, the t_1/2_ values of involved unimolecular and bimolecular reactions are calculated by t_1/2_ = ln2/*k*_a_ and t_1/2_ = ln2/(*k*_b_·[A]), respectively, where *k*_a_ and *k*_b_ represent the unimolecular and bimolecular rate constants, and [A] denotes the concentration of reactive species (e.g., OH^−^ or H^+^). $R_{H+}^{\mathrm{DMP}}1-5$, $R_{H+}^{\mathrm{DMP}}2-2$, and $R_{\mathrm{OH}-}^{\mathrm{DMP}}1-1$ are the rate-limiting steps in $R_{H+}^{\mathrm{DMP}}1$, $R_{H+}^{\mathrm{DMP}}2$, and $R_{\mathrm{OH}-}^{\mathrm{DMP}}1$, respectively, with the t_1/2_ values of 4.75 × 10^12^, 4.75 × 10^5^ and 8.46 × 10^3^ s. The estimated t_1/2_ of $R_{\mathrm{OH}-}^{\mathrm{DMP}}1-1$ is consistent with a previous study which calculated the t_1/2_ of 5.5 × 10^3^ s for OH^−^-mediated hydrolysis of DMP at pH = 7 [54]. Furthermore, the t_1/2_ value of $R_{\mathrm{OH}-}^{\mathrm{DMP}}1-1$ is 9 and 2 orders of magnitude lower than those of $R_{H+}^{\mathrm{DMP}}1-5$ and $R_{H+}^{\mathrm{DMP}}2-2$, respectively (Table S2), indicating the dominant role of OH^−^-mediated hydrolysis of DMP. Similarly, the OH^−^-mediated pathways are the most kinetically favorable for the hydrolysis of the other three PAEs. Hence, the OH^−^-mediated hydrolysis reactions are the dominant hydrolysis reaction pathways of PAEs in the aqueous phase, forming four neutral hydrolysates, i.e., MMP, monoethyl phthalate (MEP), monopropyl phthalate (MPP), and monobutyl phthalate (MBP).

**Figure 2:**
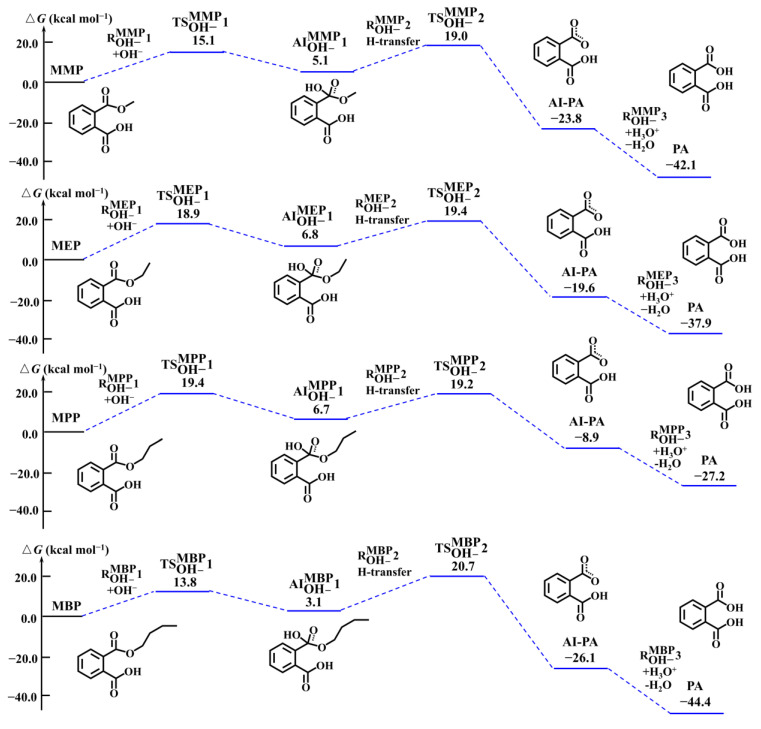
The PESs and corresponding structures of the subsequent hydrolysis reactions for MMP, MEP, MPP, and MBP.

Considering the other -COO(R) functional groups, further OH^−^-mediated hydrolysis reactions of MMP, MEP, MPP, and MBP are investigated, and the PESs are presented in Figure 2. The OH^−^-mediated hydrolysis reaction pathways of each hydrolysate involve three elementary reactions: (i) OH^−^-addition to the target hydrolysates, (ii) intramolecular proton transfer reaction, and (iii) subsequent neutralization, resulting in the formation of phthalic acid (PA). The processes (i) and (ii) proceed via the corresponding TS, with Δ*G*^‡^ values ranging from 12.5 to 19.4 kcal mol^−1^, and the corresponding *k* values range from 60 to 8.0 × 10^5^ M^−1^ s^−1^. Hence, phthalate monoesters (MMP, MEP, MPP, and MBP) and PA are the major hydrolysates of four PAEs.

### 3.2. Hydrolysate-Mediated Dimerization of GL and MG

**Figure 3:**
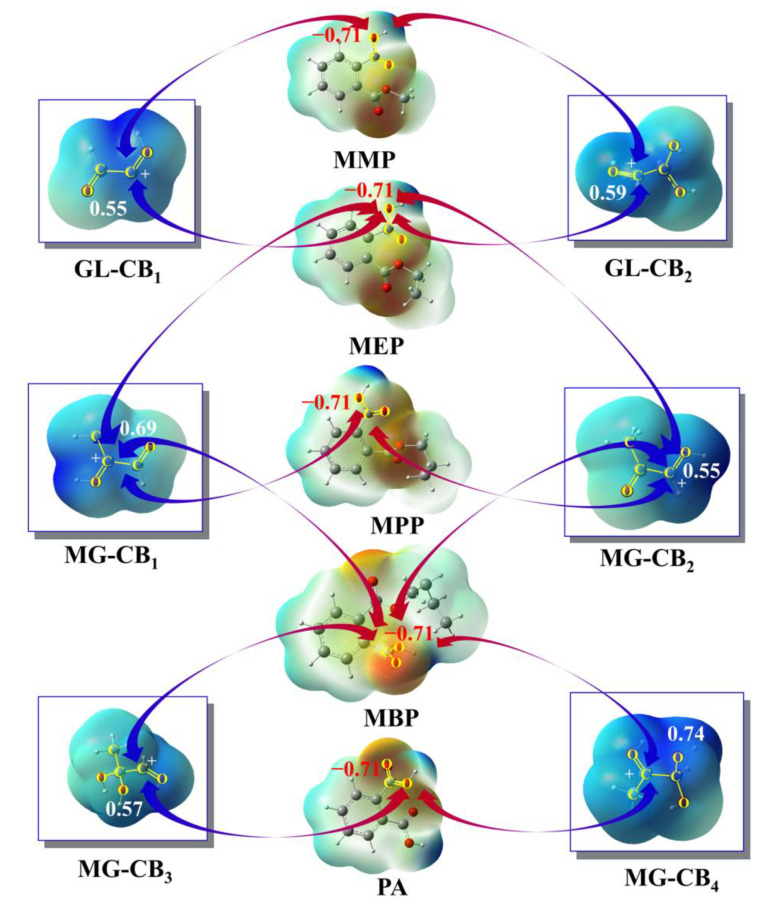
The driving force for the oligomerization of five hydrolysates with GL-CBs and MG-CBs: Electrostatic potential (ESP) maps of five hydrolysates, GL-CBs and MG-CBs, where blue and red colors indicate maximum positive and negative charge densities, respectively. The numbers in the figure represent the NPA charge values of the reaction sites (in e). The blue and red arrows point to the positive centers of the carbenium centers of CBs and the negative centers of hydrolysis products, respectively.

**Figure 4:**
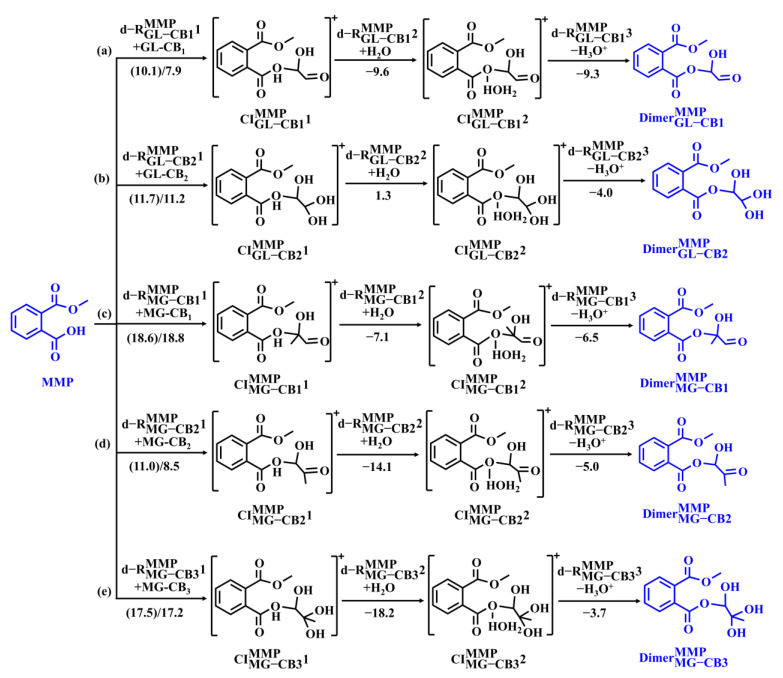
The PESs of the association reactions of MMP with (**a**) GL-CB_1_, (**b**) GL-CB_2_, (**c**) MG-CB_1_, (**d**) MG-CB_2_, and (**e**) MG-CB_3_. The number denotes the values of ∆*G*_r_ and Δ*G*^‡^ (in brackets) for each reaction step (in kcal mol^−1^).

The natural population analysis (NPA) was conducted to evaluate the reactivity of the five hydrolysates (MMP, MEP, MPP, MBP, and PA) using the natural bond orbital (NBO) method. As depicted in Figure 3, O atoms of the hydroxyl groups in these hydrolysates exhibit negative charge characteristics with the NPA charge values of −0.71 e, implying that these hydrolysates are likely to nucleophilic attack electrophilic species. For the aqueous solution containing glyoxal (GL) and methylglyoxal (MG), carbenium ions are broadly and rapidly formed [43,55], representing a class of electrophilic species. Herein, first-generation carbenium ions (CBs) are selected for the nucleophilic reactions with hydrolysates mentioned above to engage in GL/MG oligomerization. The PESs for the possible nucleophilic pathways of five hydrolysates with GL/MG-CBs are shown in Figure 4 and Figures S9–S13. The optimized geometries of all SPs in the dimerization pathways are shown in Figures S14 and S15.

Nucleophilic addition of MMP to five GL/MG-CBs proceeds via three-step reactions in sequence (Figure 4): (i) nucleophilic attack of MMP to CBs (${d-R}_{\mathrm{CBs}}^{\mathrm{MMP}}1$), (ii) hydration reaction (${d-R}_{\mathrm{CBs}}^{\mathrm{MMP}}2$), and (iii) deprotonation reaction (${d-R}_{\mathrm{CBs}}^{\mathrm{MMP}}3$). As shown in Figure 4a, for the MMP + GL-CB_1_ reaction pathway (${d-R}_{\mathrm{GL}-\mathrm{CB}1}^{\mathrm{MMP}}$), nucleophilic attack of MMP to GL-CB_1_ (${d-R}_{\mathrm{GL}-\mathrm{CB}1}^{\mathrm{MMP}}1$) forms a cationic intermediate (CI) ${\mathrm{CI}}_{\mathrm{GL}-\mathrm{CB}1}^{\mathrm{MMP}}1$, with the Δ*G*^‡^ and Δ*G*_r_ values of 10.1 and 7.9 kcal mol^−1^, respectively. Subsequently,${\mathrm{CI}}_{\mathrm{GL}-\mathrm{CB}1}^{\mathrm{MMP}}1$ undergoes barrierless hydration (${d-R}_{\mathrm{GL}-\mathrm{CB}1}^{\mathrm{MMP}}2$) to form ${\mathrm{CI}}_{\mathrm{GL}-\mathrm{CB}1}^{\mathrm{MMP}}2$, with the Δ*G*_r_ value of −9.6 kcal mol^−1^. The subsequent deprotonation reaction (${d-R}_{\mathrm{GL}-\mathrm{CB}1}^{\mathrm{MMP}}3$) is also barrierless, forming an ester-like dimer (${\mathrm{Dimer}}_{\mathrm{GL}-\mathrm{CB}1}^{\mathrm{MMP}}$), with the Δ*G*_r_ value of −9.3 kcal mol^−1^. Similarly, an ester-like dimer (${\mathrm{Dimer}}_{\mathrm{GL}-\mathrm{CB}2}^{\mathrm{MMP}}$) is also yielded from the nucleophilic reaction of MMP with GL-CB_2_ (Figure 4b), which is another first-generation CB in the GL system. However, GL-CB_2_ exhibits a more positive charge character at the carbenium site than GL-CB_1_ (Figure S2), but the Δ*G*^‡^ value of ${d-R}_{\mathrm{GL}-\mathrm{CB}2}^{\mathrm{MMP}}2$ is larger than that of ${d-R}_{\mathrm{GL}-\mathrm{CB}1}^{\mathrm{MMP}}1$. It implies that the reactivity of the nucleophilic reaction is dominantly affected by steric effect rather than NPA character, which is attributed to the presence of two additional hydroxyl groups in GL-CB_2_.

Reactions of MMP with MG-CB_1–3_ (${d-R}_{\mathrm{MG}-\mathrm{CB}1}^{\mathrm{MMP}}$, ${d-R}_{\mathrm{MG}-\mathrm{CB}2}^{\mathrm{MMP}}$, and ${d-R}_{\mathrm{MG}-\mathrm{CB}3}^{\mathrm{MMP}}$) proceed via the three-step pattern, while the nucleophilic reaction of MG-CB_4_ with MMP (${d-R}_{\mathrm{MG}-\mathrm{CB}4}^{\mathrm{MMP}}$,) is hindered from the structural and thermodynamical perspectives (detailed in Section S2.1 and Figure S13 within the Supplementary Material). The corresponding PESs are shown in Figure 4c–e. TSs are identified in ${d-R}_{\mathrm{MG}-\mathrm{CB}1}^{\mathrm{MMP}}1$, ${d-R}_{\mathrm{MG}-\mathrm{CB}2}^{\mathrm{MMP}}1$, and ${d-R}_{\mathrm{MG}-\mathrm{CB}3}^{\mathrm{MMP}}1$, with the Δ*G*^‡^ values ranging from 11.0 to 18.6 kcal mol^−1^. The smallest Δ*G*^‡^ (11.0 kcal mol^−1^) for ${d-R}_{\mathrm{MG}-\mathrm{CB}2}^{\mathrm{MMP}}1$ is attributed to the minimal steric hindrance of MG-CB_2_ due to the absence of methyl and vicinal hydroxyl groups. Subsequent hydration and deprotonation reactions are all barrierless, with the Δ*G*_r_ values in the range of −18.2~−7.1 and −6.5~−3.7 kcal mol^−1^, respectively, ultimately forming three ester-like dimers.

The reactions of the other four hydrolysates (MEP, MPP, MBP, and PA) with GL/MG-CBs are also investigated (Figures S9–S12). Similar to the dimerization of MMP with MG-CB_4_, the reactions of the other four hydrolysates with MG-CB_4_ are also difficult to occur. These hydrolysates react with GL-CB_1–2_ and MG-CB_1–3_ by following the three-step pattern, finally forming twenty ester-like dimers. For example, nucleophilic attack of MEP to GL-CB_1_ (${d-R}_{\mathrm{GL}-\mathrm{CB}1}^{\mathrm{MEP}}1$) forms ${\mathrm{CI}}_{\mathrm{GL}-\mathrm{CB}1}^{\mathrm{MEP}}1$, with the Δ*G*^‡^ value of 8.9 kcal mol^−1^. Subsequently,${\mathrm{CI}}_{\mathrm{GL}-\mathrm{CB}1}^{\mathrm{MEP}}1$ undergoes barrierless hydration (${d-R}_{\mathrm{GL}-\mathrm{CB}1}^{\mathrm{MEP}}2$) and deprotonation (${d-R}_{\mathrm{GL}-\mathrm{CB}1}^{\mathrm{MEP}}3$) to form an ester-like dimer (${\mathrm{Dimer}}_{\mathrm{GL}-\mathrm{CB}1}^{\mathrm{MEP}}$), with the total Δ*G*_r_ value of −6.1 kcal mol^−1^. Given the barrierless characters of the second and third steps, $d-R_{\mathrm{GL}-\mathrm{CBs}}^{\mathrm{HD}}1$ and $d-R_{\mathrm{MG}-\mathrm{CBs}}^{\mathrm{HD}}1$ (HD denotes MEP, MPP, MBP, and PA) pathways, with the Δ*G*^‡^ values ranging from 8.9 to 15.4 and 9.7 to 18.4 kcal mol^−1^, respectively, are the rate-limiting steps in the ester-like dimer formation in the GL and MG reaction systems.

The rate constants of ${d-R}_{\mathrm{CBs}}^{\mathrm{HD}}$ are estimated using the rate-limiting steps (${d-R}_{\mathrm{CBs}}^{\mathrm{HD}}$1) to evaluate the kinetics of ester-like dimer formation from GL/MG-CBs with hydrolysates (Tables S3 and S4). For the GL + hydrolysates reaction system, the *k* values of ${d-R}_{\mathrm{GL}-\mathrm{CB}}^{\mathrm{HD}}1$ are in the range of 5.28 × 10^4^~1.12 × 10^9^ M^−1^ s^−1^, and ${d-R}_{\mathrm{GL}-\mathrm{CB}1}^{\mathrm{MEP}}$ is the dominant pathway with the largest *k* value of 1.12 × 10^9^ M^−1^ s^−1^. As for the MG-CBs + hydrolysates reaction system, the *k* values of ${d-R}_{\mathrm{MG}-\mathrm{CB}}^{\mathrm{HD}}1$ are in the range of 2.33 × 10^2^~5.58 × 10^8^ M^−1^ s^−1^, and ${d-R}_{\mathrm{MG}-\mathrm{CB}2}^{\mathrm{MBP}}$ is the dominant pathway with the largest *k* value of 5.58 × 10^8^ M^−1^ s^−1^. The larger rate constants suggest that the PAE-mediated dimerization of GL is more kinetically favorable than that of MG. Additionally, the *k* values for GL and MG dimerization in the presence of PAE reached up to 1.12 × 10^9^ M^−1^ s^−1^, which are comparable to those of their self-oligomerization (10^9^–10^10^ M^−1^ s^−1^) [34,43], indicating that GL and MG oligomerization in the presence of PAEs play a non-negligible role in SOA formation.

### 3.3. Subsequent Trimerization of Ester-like Dimers

**Figure 5:**
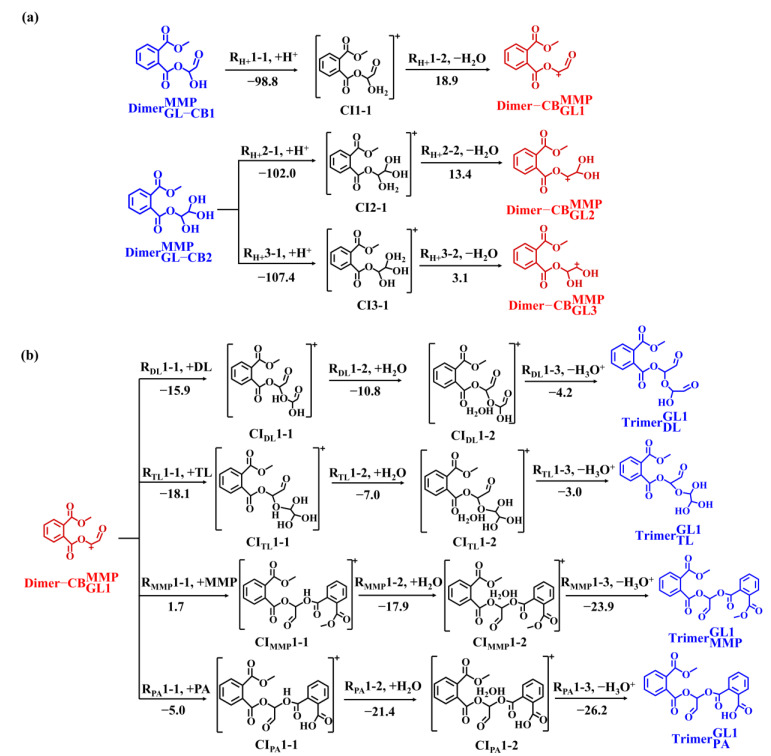
The trimerization pathways of ${\mathrm{Dimer}}_{\mathrm{GL}-\mathrm{CB}1}^{\mathrm{MMP}}$ and ${\mathrm{Dimer}}_{\mathrm{GL}-\mathrm{CB}2}^{\mathrm{MMP}}$ (**a**) protonation of ${\mathrm{Dimer}}_{\mathrm{GL}-\mathrm{CB}1}^{\mathrm{MMP}}$ and ${\mathrm{Dimer}}_{\mathrm{GL}-\mathrm{CB}2}^{\mathrm{MMP}}$, and (**b**) subsequent association reactions of ${\mathrm{Dimer}-\mathrm{CB}\;}_{\mathrm{GL}-\mathrm{CB}1}^{\mathrm{MMP}}$. The number is the Δ*G*_r_ in each reaction (in kcal mol^−1^).

As discussed above, there is the formation of twenty-five ester-like dimers in the PAE-mediated dimerization of GL/MG, which have the potential to undergo further oligomerization due to the presence of hydroxyl groups (Figures S14 and S15). Herein, we take ${\mathrm{Dimer}}_{\mathrm{GL}-\mathrm{CB}1}^{\mathrm{MMP}}$ and ${\mathrm{Dimer}}_{\mathrm{GL}-\mathrm{CB}2}^{\mathrm{MMP}}$ as the representatives of ester-like dimers to investigate the subsequent trimerization because the hydrolysis reaction of DMP yields MMP with the highest *k* value and the *k* values for MMP-mediated dimerization in the GL reaction system are higher than those of the MG system. The PESs for the subsequent pathways of ${\mathrm{Dimer}}_{\mathrm{GL}-\mathrm{CB}1}^{\mathrm{MMP}}$ and ${\mathrm{Dimer}}_{\mathrm{GL}-\mathrm{CB}2}^{\mathrm{MMP}}$ are presented in Figure 5a. For ${\mathrm{Dimer}}_{\mathrm{GL}-\mathrm{CB}1}^{\mathrm{MMP}}$, the protonation occurs at the -OH group (R_H+_1-1) to form CI1-1, which subsequently undergoes dehydration (R_H+_1-2), with the total Δ*G*_r_ value of −79.9 kcal mol^−1^, to yield $\mathrm{Dimer}-{\mathrm{CB}}_{\mathrm{GL}1}^{\mathrm{MMP}}$. Similarly, ${\mathrm{Dimer}}_{\mathrm{GL}-\mathrm{CB}2}^{\mathrm{MMP}}$ proceeds via protonation (R_H+_2-1 and R_H+_3-1), and dehydration (R_H+_2-2 and R_H+_3-2), to yield $\mathrm{Dimer}-{\mathrm{CB}}_{\mathrm{GL}2}^{\mathrm{MMP}}$ and $\mathrm{Dimer}-{\mathrm{CB}}_{\mathrm{GL}3}^{\mathrm{MMP}}$, with the total Δ*G*_r_ values of −88.6 and −104.3 kcal mol^−1^, respectively. In conclusion, three Dimer-CBs are generated via sequential protonation and dehydration of ${\mathrm{Dimer}}_{\mathrm{GL}-\mathrm{CB}1}^{\mathrm{MMP}}$ and ${\mathrm{Dimer}}_{\mathrm{GL}-\mathrm{CB}2}^{\mathrm{MMP}}$.

As shown in Figure 5b and Figure S16, these three Dimer-CBs are feasible to be attacked by nucleophilic species such as Diol (DL), Tetrol (TL), MMP, and PA because of positive charge centers to form the corresponding ester-like trimers (${\mathrm{Trimer}}_{\mathrm{DL}\;}^{\mathrm{GL}1}$, ${\mathrm{Trimer}}_{\mathrm{TL}\;}^{\mathrm{GL}1}$, ${\mathrm{Trimer}}_{\mathrm{MMP}}^{\mathrm{GL}1}$, ${\mathrm{Trimer}}_{\mathrm{PA}\;}^{\mathrm{GL}1}$, ${\mathrm{Trimer}}_{\mathrm{DL}\;}^{\mathrm{GL}2}$, ${\mathrm{Trimer}}_{\mathrm{TL}\;}^{\mathrm{GL}2}$, ${\mathrm{Trimer}}_{\mathrm{MMP}}^{\mathrm{GL}2}$, ${\mathrm{Trimer}}_{\mathrm{PA}\;}^{\mathrm{GL}2}$, ${\mathrm{Trimer}}_{\mathrm{DL}\;}^{\mathrm{GL}3}$, ${\mathrm{Trimer}}_{\mathrm{TL}\;}^{\mathrm{GL}3}$, ${\mathrm{Trimer}}_{\mathrm{MMP}}^{\mathrm{GL}3}$, and ${\mathrm{Trimer}}_{\mathrm{PA}\;}^{\mathrm{GL}3}$). For example, the positive charge center of $\mathrm{Dimer}-{\mathrm{CB}}_{\mathrm{GL}1}^{\mathrm{MMP}}$ is attacked by -OH group in DL (R_DL_1-1) to form CI_DL_1-1, with Δ*G*_r_ value of −15.9 kcal mol^−1^. It is evident from Figure 5b that the exothermicity of ${\mathrm{Dimer}-\mathrm{CB}}_{\mathrm{GL}1}^{\mathrm{MMP}}$ with DL/TL is larger than that with MMP/PA, attributable to the more negative natural charges on the O-atoms of DL and TL (Figure S2). Subsequently, the CI_DL_1-1 undergoes hydration (R_DL_1-2) and deprotonation (R_DL_1-3) to yield ${\mathrm{Trimer}}_{\mathrm{DL}\;}^{\mathrm{GL}1}$, with a total Δ*G*_r_ value of −35.9 kcal mol^−1^. In summary, twelve trimers are favorably formed via the trimerization reactions of ${\mathrm{Dimer}}_{\mathrm{GL}-\mathrm{CB}1}^{\mathrm{MMP}}$ and ${\mathrm{Dimer}}_{\mathrm{GL}-\mathrm{CB}2}^{\mathrm{MMP}}$, which implies that structurally analogous dimers generated via hydrolysate-mediated GL/MG dimerization can undergo similar trimerization to form trimers. Furthermore, the formed trimers contain hydroxyl groups critical for further oligomerization: there is one -OH group in ${\mathrm{Trimer}}_{\mathrm{DL}\;}^{\mathrm{GL}1}$ and ${\mathrm{Trimer}}_{\mathrm{PA}\;}^{\mathrm{GL}1}$, three -OH groups in ${\mathrm{Trimer}}_{\mathrm{TL}\;}^{\mathrm{GL}1}$. According to a previous study [34], the C=O group in ${\mathrm{Trimer}}_{\mathrm{MMP}\;}^{\mathrm{GL}1}$ can be transformed into two -OH groups. Hence, the characteristic of containing at least one -OH group indicates that these trimers are favorable for further oligomerization to form higher-molecular-weight oligomers.

## 4. Conclusions

Aqueous-phase oligomerization of GL and MG is the key pathway for SOA formation. However, the influence of coexisting constituents, such as emerging contaminants (ECs), on these oligomerization mechanisms remains unclear. In this study, the aqueous-phase oligomerization of GL and MG in the presence of PAEs, a typical class of ECs in urban atmospheric aerosols, was investigated by using DFT calculations. Our findings indicate that the oligomerization of GL/MG cannot be directly regulated by the four target PAEs (i.e., DMP, DEP, DPP, and DBP) but can be initiated by hydrolysates (MMP, MEP, MPP, MBP, and PA) of PAEs. These hydrolysates are readily formed by OH^−^-mediated hydrolysis reactions of the target PAEs, subsequently participating in the oligomerization of GL/MG attributable to the enhancement of the reactivity by introducing additional -OH groups. The hydrolysate-mediated oligomerization of GL/MG proceeds via three-step reactions, including (i) nucleophilic attack of -OH groups to CBs, (ii) hydration, and (iii) deprotonation. Among these reactions, the nucleophilic attack of hydrolysates to CBs is the rate-limiting step to form twenty-five ester-like dimers. The PAE-mediated dimerization of GL is more kinetically favorable than that of MG, which is attributable to the greater reactivity of GL-CBs caused by smaller steric hindrances. Subsequently, these dimers repeat the three-step reactions to form trimers, and the barrierless characters indicate the favorable formation of trimers.

The oligomerization of GL/MG in the presence of PAEs exhibits a non-negligible contribution to SOA, as evidenced by two aspects. On the one hand, the half-life (t_1/2_) of OH^−^-initiated transformation is estimated to be 2.4–42.0 h at pH = 6 using the formula t_1/2_ = ln2/(*k*_OH-11_·[OH^−^]) [54], where *k*_OH-11_ is the *k* value of rate-limiting step in OH^−^-mediated hydrolysis reactions. It suggests that the hydrolysates containing -OH groups are preferred forms of PAEs under weakly acidic conditions. On the other hand, the identified trimers exhibit high-molecular-weight characters with low volatility (detailed in Section S2.2 within the Supplementary Material), which undergo successive oligomerization through the proposed three-step reactions mechanism due to nucleophilic -OH groups. Therefore, greater attention should be paid to SOA formation from the PAE-mediated GL/MG oligomerization in weakly acidic aerosols, particularly those with a pH value approaching neutrality. This study conducted a systematic exploration of the oligomerization of α-dicarbonyl in the presence of PAEs, supplementing the potential mechanisms of emerging contaminants involved in the SOA formation through the oligomerization of dicarbonyls. Our results provide the kinetic and mechanistic data for the inclusion of the GL and MG oligomerization in the presence of PAEs in atmospheric models, helping improve the model performance in evaluating the SOA budget. Future studies are necessary to assess the impacts of PAEs on SOA formation from α-dicarbonyls using chemical transport models, with the consideration of its emission inventory, chemistry, and transport. Additionally, it provides a new insight into SOA formation from traditional OVOCs, considering the influence of more ECs.

## Data Availability

All raw data can be provided by the corresponding author upon request.

## Supplementary Materials

The following supporting information can be downloaded at: https://www.mdpi.com/article/10.3390/toxics13040272/s1, Figure S1: The optimized geometries of DMP, DEP, DPP, and DBP at the level of M06-2X/6-311G(d,p); Figure S2: The NPA charge values of key species obtained at the M06-2X/6-311G(d,p) level; Figure S3: The PESs for the direct hydrolysis reactions of (a) DMP, (b) DEP, (c) DPP, and (d) DBP obtained at the level of M06-2X//M06-2X; Figure S4: The PESs for the indirect hydrolysis reactions of (a) DEP, (b) DPP, and (c) DBP obtained at the level of M06-2X//M06-2X; Figure S5: The optimized structures of key species in the DMP hydrolysis reaction at the level of M06-2X/6-311G(d,p); Figure S6: The optimized structures of key species in the DEP hydrolysis reaction at the level of M06-2X/6-311G(d,p); Figure S7: The optimized structures of key species in the DPP hydrolysis reaction at the level of M06-2X/6-311G(d,p); Figure S8: The optimized structures of key species in the DBP hydrolysis reaction at the level of M06-2X/6-311G(d,p); Figure S9: The PESs of the association reactions of MEP and GL-CBs and MG-CBs; Figure S10: The PESs of the association reactions of MPP and GL-CBs and MG-CBs; Figure S11: The PESs of the association reactions of MBP and GL-CBs and MG-CBs; Figure S12: The PESs of the association reactions of PA and GL-CBs and MG-CBs; Figure S13: The pointwise potential curve scanning of the association reactions between MG-CB4 and MMP, MEP, MPP, MBP, and PA, and the corresponding geometries; Figure S14: The geometries of the TSs and ester-like dimer for the oligomerization reaction between GL-CBs (denoted as GL-CB1 and GL-CB2) and five hydrolysis products (MMP, MEP, MPP, MBP, and PA) obtained at the level of the M06-2X/6-311G(d,p); Figure S15: The geometries of the TSs and ester-like dimer for the oligomerization reaction between MG-CBs (denoted as MG-CB1, MG-CB2, and GL-CB3) and five hydrolysis products (MMP, MEP, MPP, MBP, and PA) obtained at the level of the M06-2X/6-311G(d,p); Figure S16: The subsequent association reactions of ${\mathrm{Dimer}-\mathrm{CB}\;}_{\mathrm{GL}-\mathrm{CB}2}^{\mathrm{MMP}}$ and ${\mathrm{Dimer}-\mathrm{CB}\;}_{\mathrm{GL}-\mathrm{CB}3}^{\mathrm{MMP}}$; Table S1: The *k* values for the association reactions between GL-CBs (s = 1, 2) and MMP, MEP, MPP, MBP, and PA at 298 K; Table S2: The t_1/2_ values at pH = 6 for the hydrolysis reactions of DMP, DEP, DPP and DBP at 298 K; Table S3: The *k* values for the association reactions between GL-CBs (s = 1, 2) and MMP, MEP, MPP, MBP, and PA at 298 K; Table S4: The *k* values for the association reactions between MG-CBs (s = 1, 2, 3) and MMP, MEP, MPP, MBP, and PA at 298 K. Reference [56] is cited in the supplementary materials.
