# Supplementary material for: Aqueous-Phase Reaction Mechanisms of Small α-Dicarbonyls in the Presence of Phthalate Esters
Source: Toxics. 2025 Apr 2;13(4):272. doi: 10.3390/toxics13040272 (PMC12031212; doi:10.3390/toxics13040272)
Supplement: Supplementary file 1 [file toxics-13-00272-s001.zip › toxics-3532629-supplementary.pdf]

Supplement of

Aqueous-phase Reaction Mechanisms of Small α-Dicarbonyls in the Presence of Phthalate Esters

Wenjian Li [1,2], Qiuju Shi [1,2], Jiaxin Wang [1,2], Ruize Ma [1,2], Yanpeng Gao [1,2] and Yuemeng Ji [1,2,*]

1 Guangdong-Hong Kong-Macao Joint Laboratory for Contaminants Exposure and Health, Guangdong Key Laboratory of Environmental Catalysis and Health Risk Control, Institute Environmental Health and Pollution Control, Guangdong University of Technology, Guangzhou 510006, China

2 Guangdong Basic Research Center of Excellence for Ecological Security and Green Development, Key Laboratory of City Cluster Environmental Safety and Green Development of the Ministry of Education, School of Environmental Science and Engineering, Guangdong University of Technology, Guangzhou 510006, China

* Correspondence: jiym@gdut.edu.cn (Y. Ji).

S1. Methodology

S1.1 Detailed description of rate constants

The rate constant (k) for reactions with a $\Delta G^{\ddagger}$ value was calculated using Conventional Transition State theory (TST) as follows [1,2]:

$$k_t = \sigma \frac{k_B T}{h} \exp(\frac{-\Delta G^{\ddagger}}{RT}) \quad \text{(S1)}$$

where h and k_B are the Planck and Boltzmann constants, respectively; $\Delta G^{\ddagger}$ denotes the the activation energy of the reaction, including thermodynamic corrections and solvent cage effects, and σ is the reaction path degeneracy.

To model the solvation dynamics within the realistic solution, the solvent cage effect proposed by Okuno [3] is integrated into the free volume theory. Gibbs free energy is corrected using the following expression:

$$\Delta G_{sol}^{FV} \cong \Delta G_{sol}^{0} - RT\{\ln[n10^{(2n-2)}] - (n-1)\} \quad \text{(S2)}$$

where ΔG_{sol}^{0} is the Gibbs free energy of the reaction in the solution and n denotes the number of molecules involved in the reaction. According to expression (S2), the cage effects in the solution lead to a decrease in the Gibbs free energy by 2.54 kcal mol^{-1} for bimolecular reactions occurring at 298.15 K. Thus, the apparent rate constant (k) obtained from the diffusion-limit effect [4] can be calculated by the formula as follows:

$$k = \frac{k_t k_d}{k_t + k_d} \quad \text{(S3)}$$

where the k_t is the thermal rate constant from expression (S1). The diffusion-limited rate constant (k_D) for a bimolecular reaction can be calculated as follows:

$$k_D = 4\pi R D_{AB} N_A \quad \text{(S4)}$$

where R is the reaction distance, N_A denotes the Avogadro number; and D_{AB} denotes the mutual diffusion coefficient of the reactants A and B, which is the sum of diffusion coefficients of reactants A and B (D_A and D_B), i.e., $D_{AB} = D_A + D_B$. D_A and D_B are estimated from the Stokes–Einstein [5] approach listed in expression (S5):

$$D = \frac{k_B T}{6\pi\eta\alpha} \quad \text{(S5)}$$

where k_B is the Boltzmann constant, T is the temperature, η denotes the viscosity of the solvent, which is water in our case ($\eta = 8.9 \times 10^{-4}$ Pa s), and α is the radius of the solute.

S2. Results and Discussion

S2.1 Nucleophilic reaction of $MG-CB_4$ with MMP

The C···O distance of the minimum point in the PES of the $MG-CB_4$ + MMP reaction system is 2.69 Å (Figure S13), which is longer than a typical C-O bond. Furthermore, the $E_{relative\ energy}$ value increases monotonically as the C···O distance decreases, indicating that the nucleophilic addition cannot occur in this system. This hindrance is likely due to the considerable steric hindrance from the methyl on the positive charge center and the vicinal hydroxyl groups in $MG-CB_4$.

S2.2 The molecular weight and volatility of trimers formed through PAE-mediated trimerization

The trimers formed through PAE-mediated trimerization can potentially contribute to SOA formation due to their high molecular weight (MW = 296 ~ 418). A recent study has shown that, for the organic matter in aerosol, the number of carbon-carbon double bonds and rings has significant influence on volatility, and the double bond equivalents (DBE) are notably inversely related to volatility [6]. Hence, in order to better evaluate the potentiality of the trimerization products of the reaction to SOA, the DBE values of different trimers are calculated using the following formula: DBE = C - H/2 + N/2 + 1. The DBE values of $\text{trimer}_{\text{MMP}}^{\text{GL1}}$, $\text{trimer}_{\text{PA}}^{\text{GL1}}$,$\text{trimer}_{\text{MMP}}^{\text{GL2}}$, $\text{trimer}_{\text{PA}}^{\text{GL2}}$, $\text{trimer}_{\text{MMP}}^{\text{GL3}}$, and $\text{trimer}_{\text{PA}}^{\text{GL3}}$ are ranging from 12 - 13, which are significantly higher than those of $\text{trimer}_{\text{DL}}^{\text{GL1}}$, $\text{trimer}_{\text{TL}}^{\text{GL1}}$, $\text{trimer}_{\text{DL}}^{\text{GL2}}$, $\text{trimer}_{\text{TL}}^{\text{GL2}}$, $\text{trimer}_{\text{DL}}^{\text{GL3}}$, and $\text{trimer}_{\text{TL}}^{\text{GL3}}$ (DBE values = 6 - 8). It suggests that the oligomerization of dimer-CBs with hydrolysates in the presence of PAEs yields larger molecular weight and more unsaturated trimers, leading to promoting SOA formation.

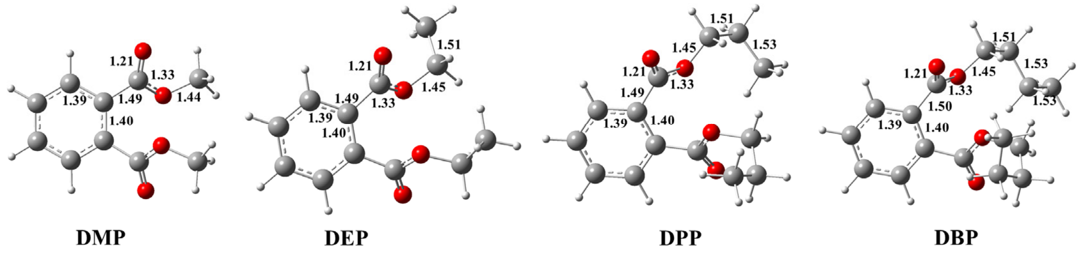

Figure S1: The optimized geometries of DMP, DEP, DPP, and DBP at the level of M06-2X/6-311G(d,p). The number is bond length (in Å) ● C; ● O; ● H

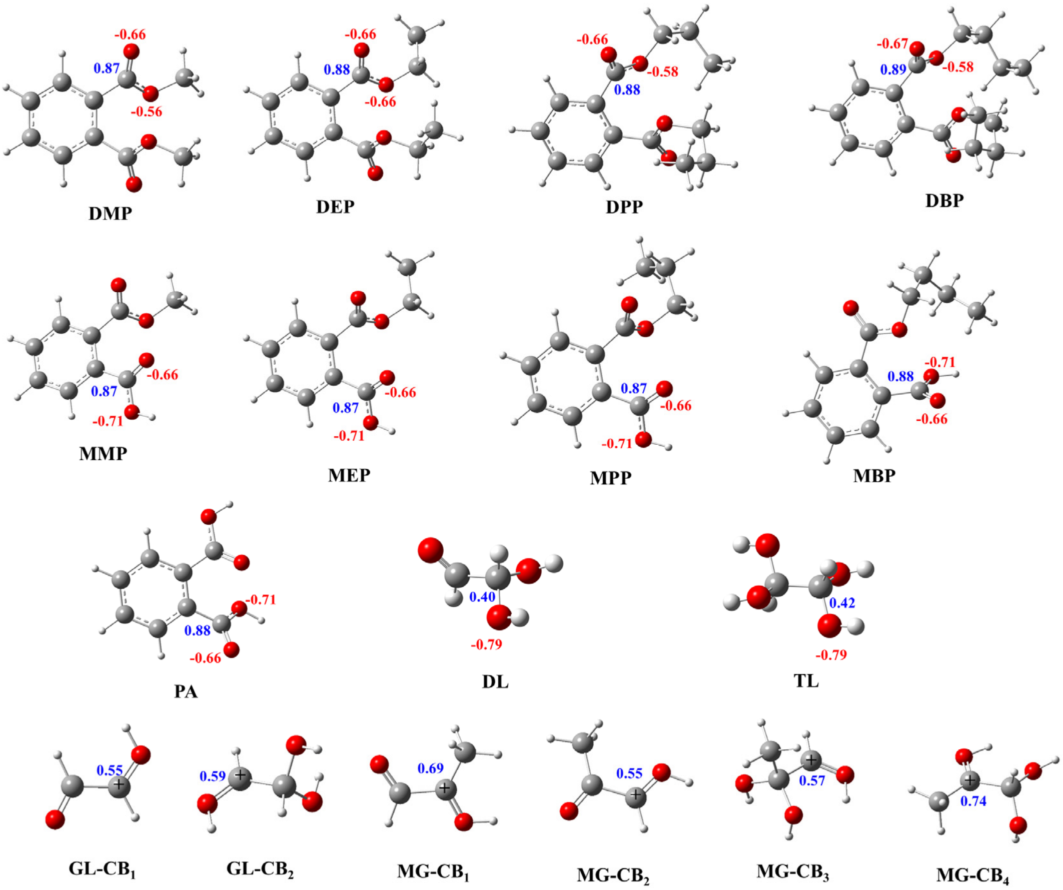

Figure S2: The NPA charge values of key species obtained at the M06-2X/6-311G(d,p) level. The numbers denote charge values (in e). ● C; ● O; ● H

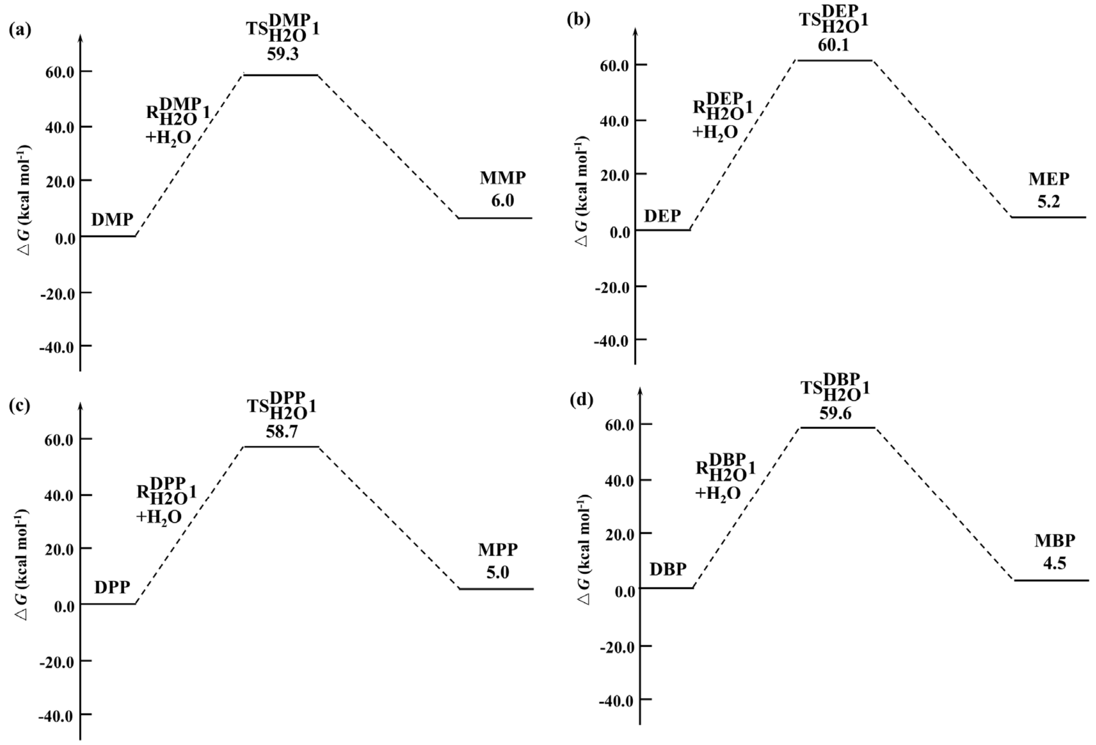

Figure S3: The PESs for the direct hydrolysis reactions of (a) DMP, (b) DEP, (c) DPP, and (d) DBP obtained at the level of M06-2X//M06-2X.

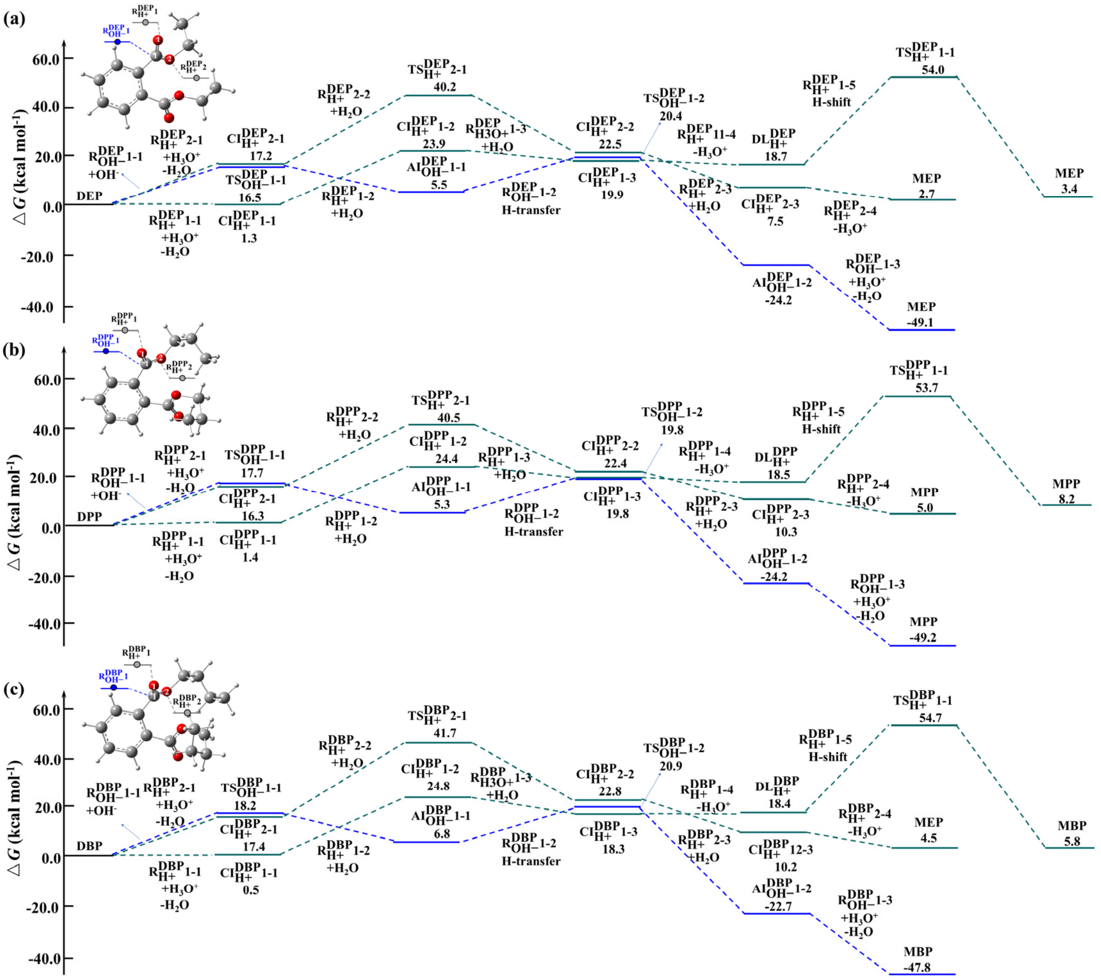

Figure S4: The PESs for the indirect hydrolysis reactions of (a) DEP, (b) DPP, and (c) DBP obtained at the level of M06-2X//M06-2X.

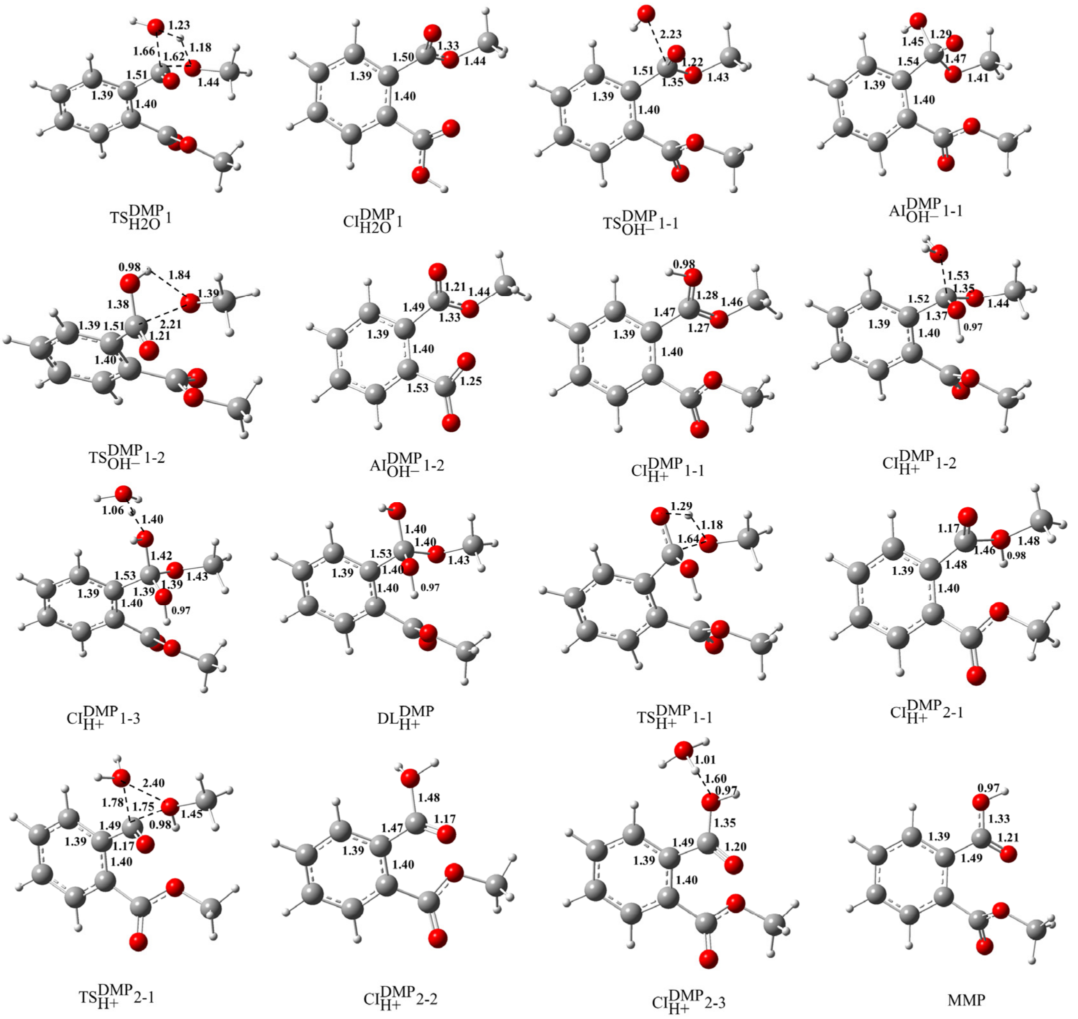

Figure S5: The optimized structures of key species in the DMP hydrolysis reaction at the level of M06-2X/6-311G(d,p). The number is bond length (in Å) C; O; H

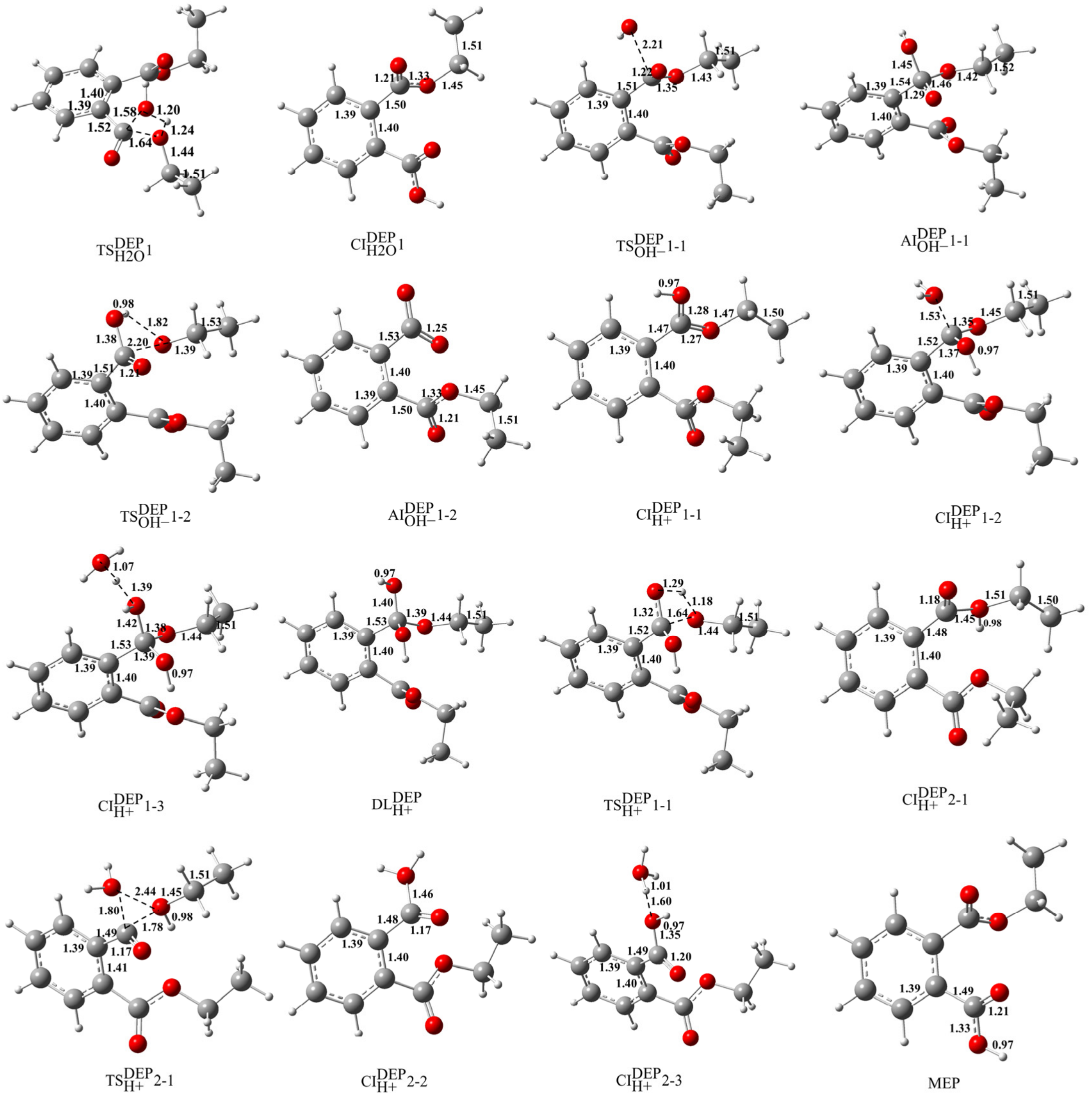

Figure S6: The optimized structures of key species in the DEP hydrolysis reaction at the level of M06-2X/6-311G(d,p). The number is bond length (in Å) C; O; H

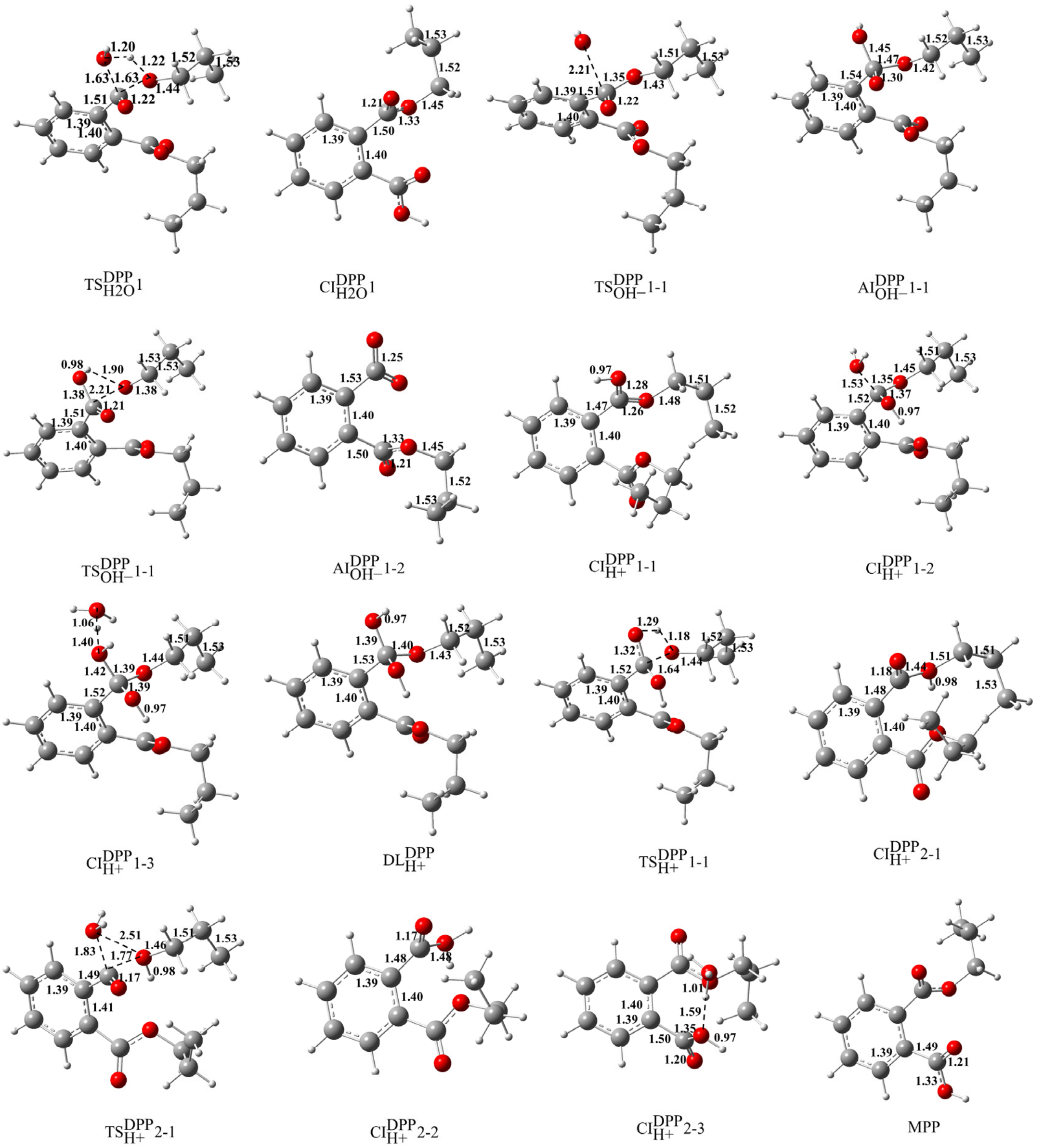

Figure S7: The optimized structures of key species in the DPP hydrolysis reaction at the level of M06-2X/6-311G(d,p). The number is bond length (in Å) C; O; H

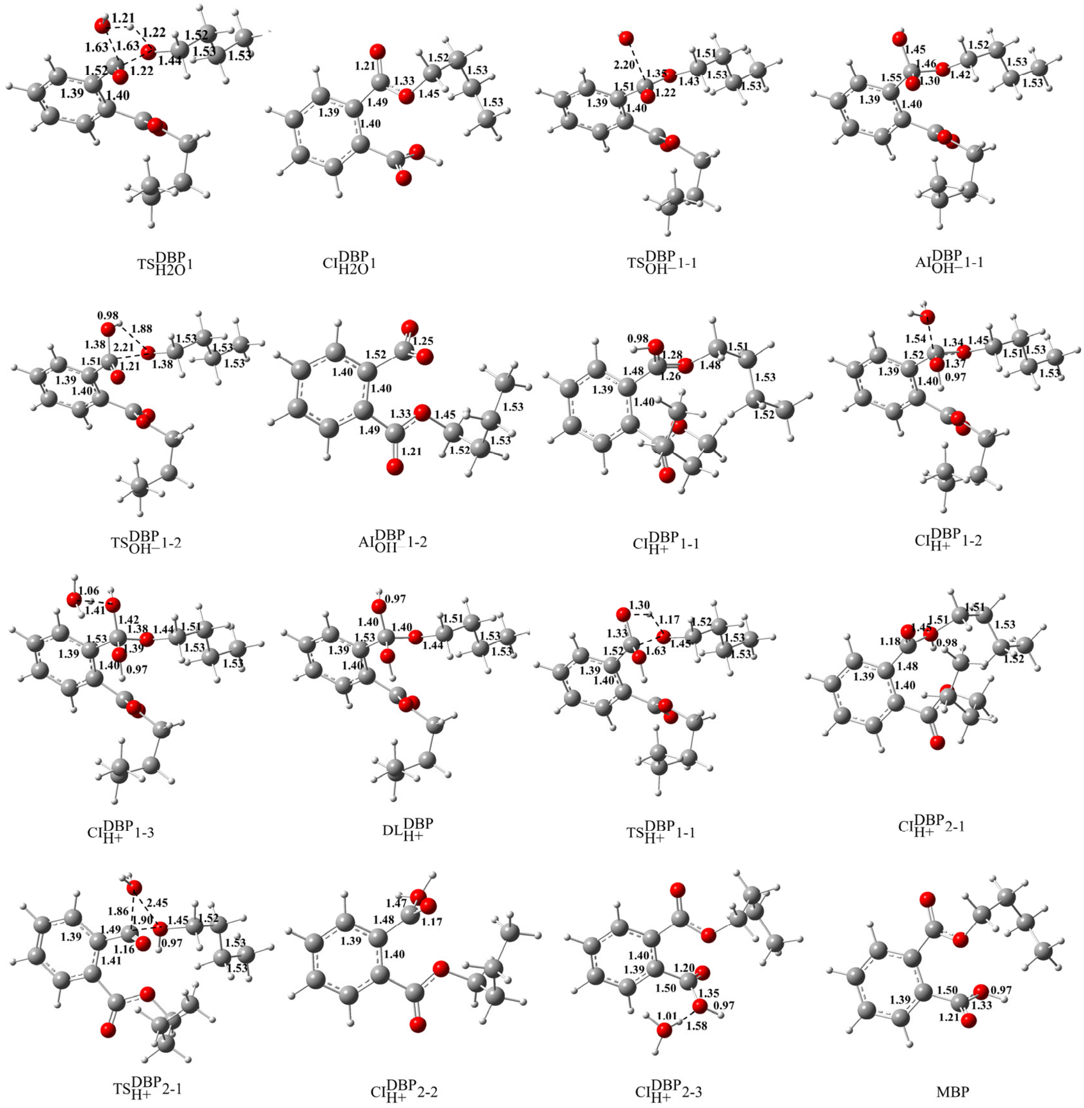

Figure S8: The optimized structures of key species in the DBP hydrolysis reaction at the level of M06-2X/6-311G(d,p). The number is bond length (in Å) ● C; ● O; ● H

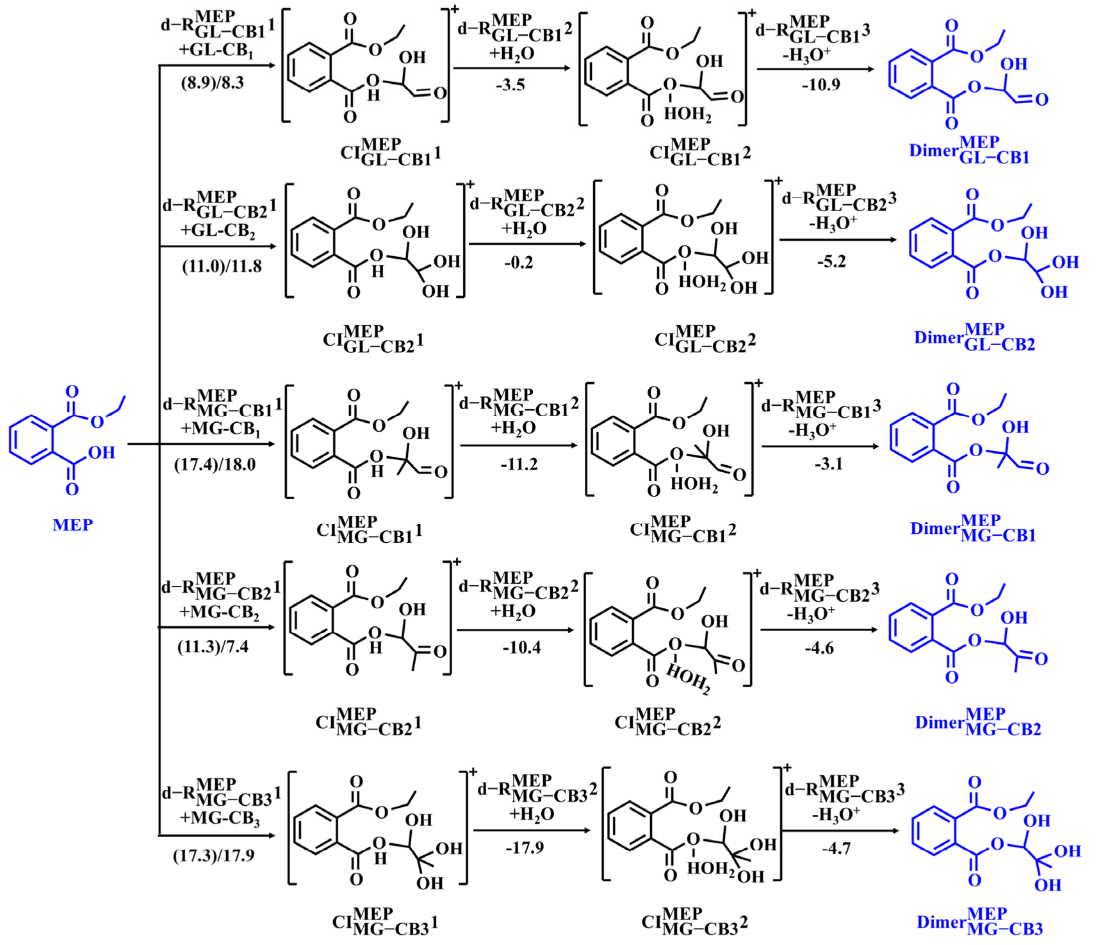

Figure S9: The PESs of the association reactions of MEP and GL-CBs and MG-CBs. The number denotes the values of ΔG_r and $\Delta G^{\ddagger}$ (in brackets) for each reaction step (in kcal mol^{-1}).

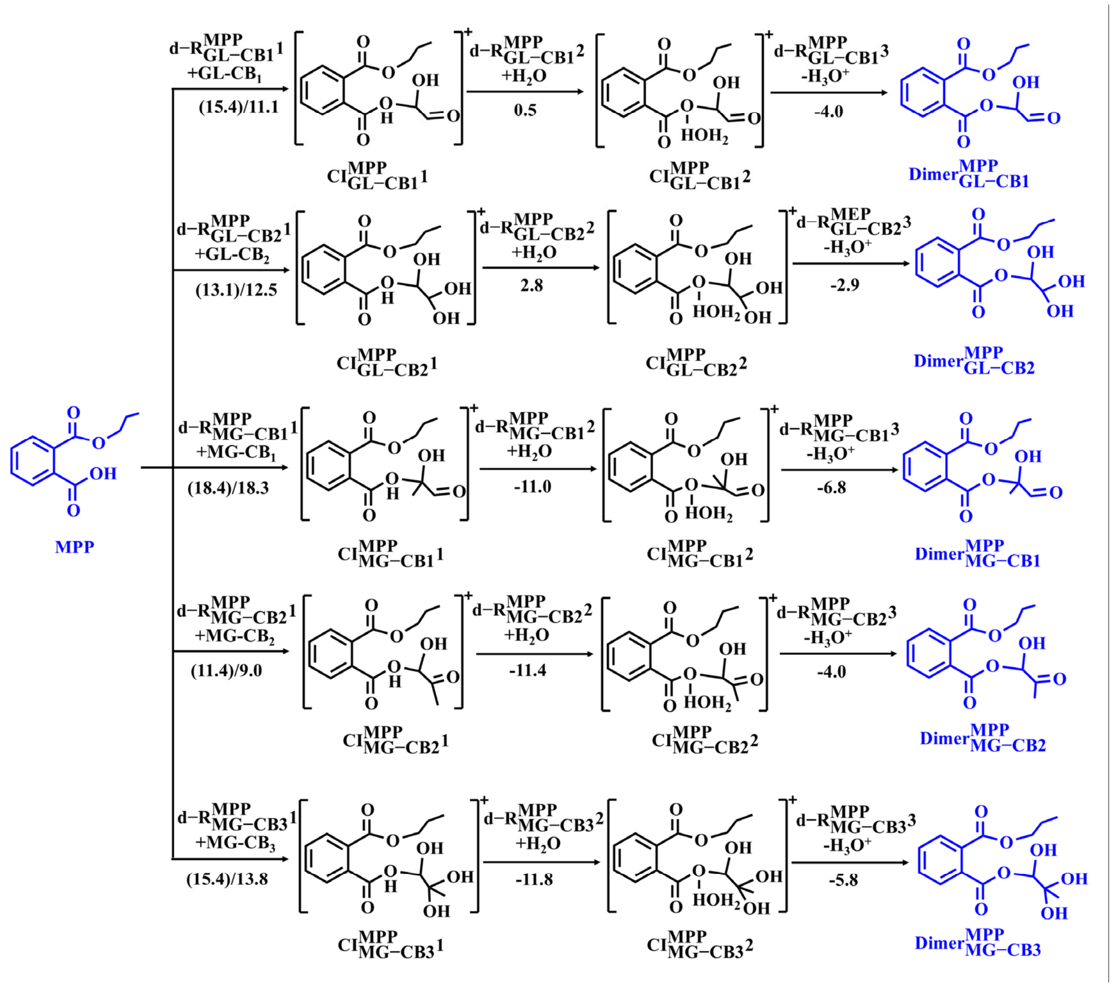

Figure S10: The PESs of the association reactions of MPP and GL-CBs and MG-CBs. The number denotes the values of ΔG_r and $\Delta G^{\ddagger}$ (in brackets) for each reaction step (in kcal mol^{-1}).

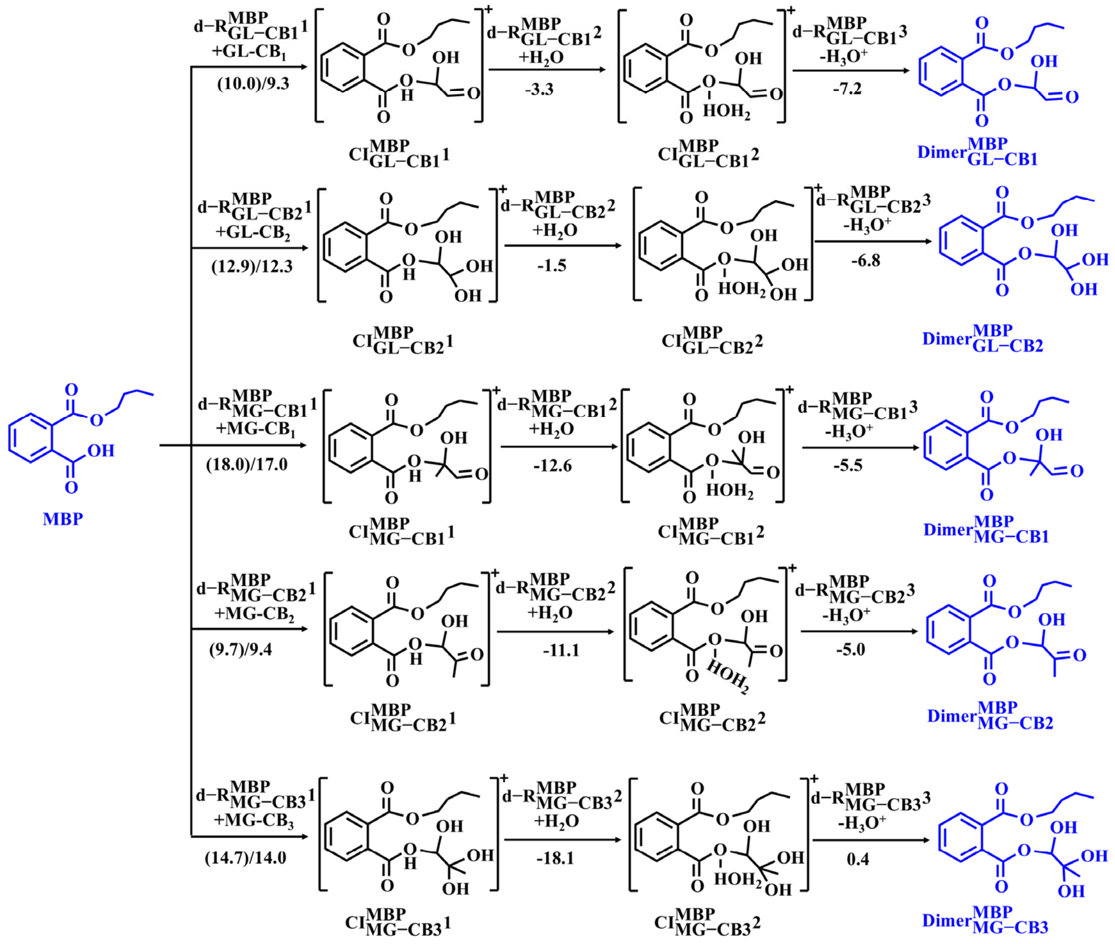

Figure S11: The PESs of the association reactions of MBP and GL-CBs and MG-CBs. The number denotes the values of ΔG_r and $\Delta G^‡$ (in brackets) for each reaction step (in kcal mol^{-1}).

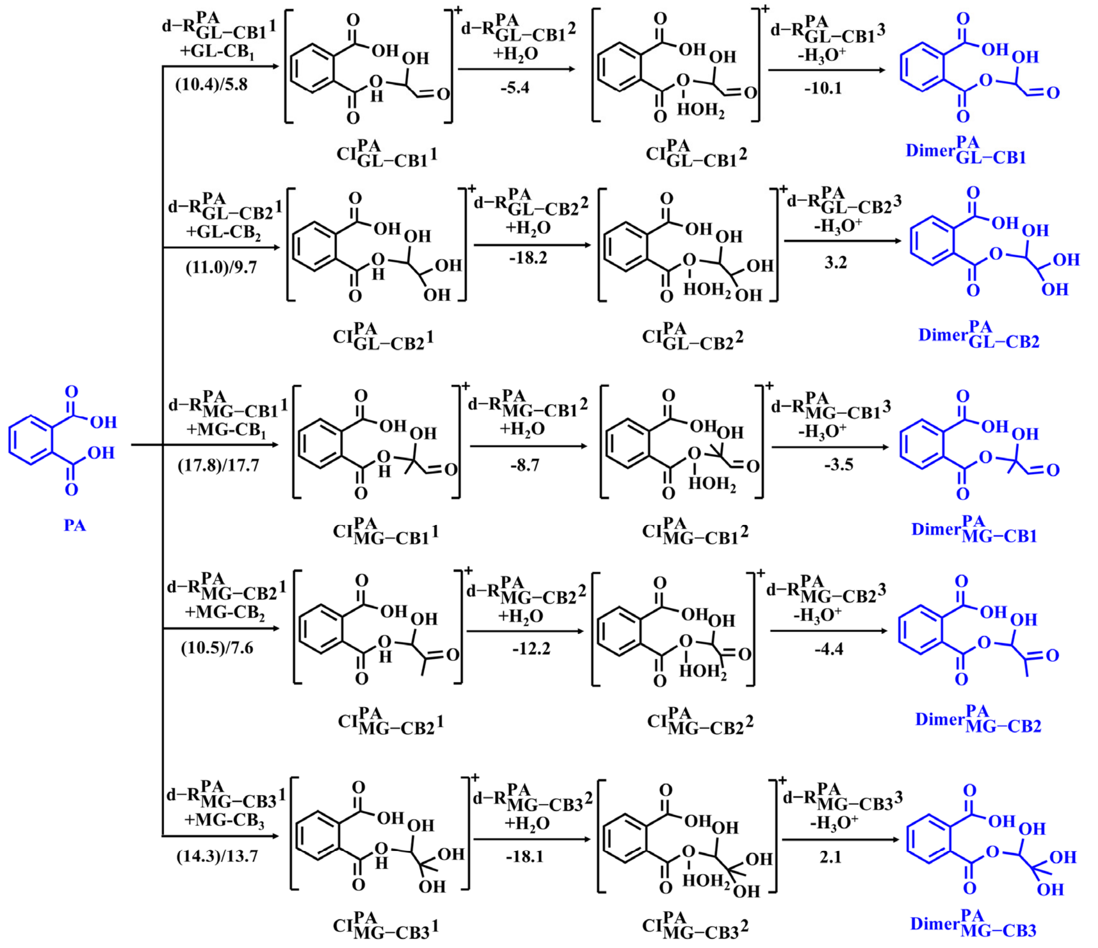

Figure S12: The PESs of the association reactions of PA and GL-CBs and MG-CBs. The number denotes the values of ΔG_r and $\Delta G^{\ddagger}$ (in brackets) for each reaction step (in kcal mol^{-1}).

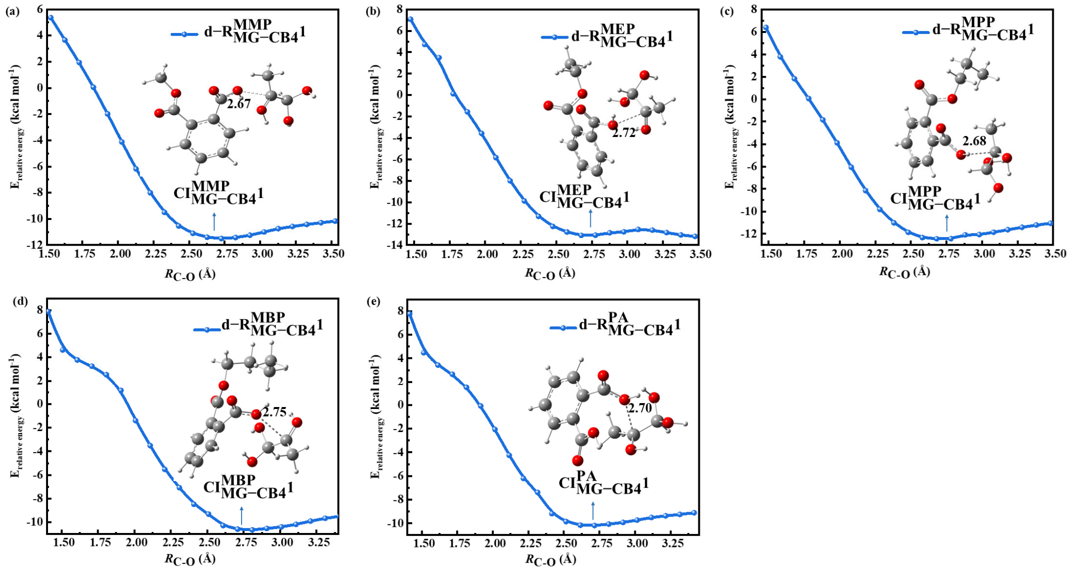

Figure S13: The pointwise potential curve scanning of the association reactions between MG-CB4 and MMP, MEP, MPP, MBP, and PA, and the corresponding geometries. R_{C-O} represents the distance (in Å) between the positive charge center C atom in MG-CB4 and hydroxyl O atom in carbonxyl group of (a) MMP, (b) MEP, (c) MPP, (d) MBP,

and (e) PA

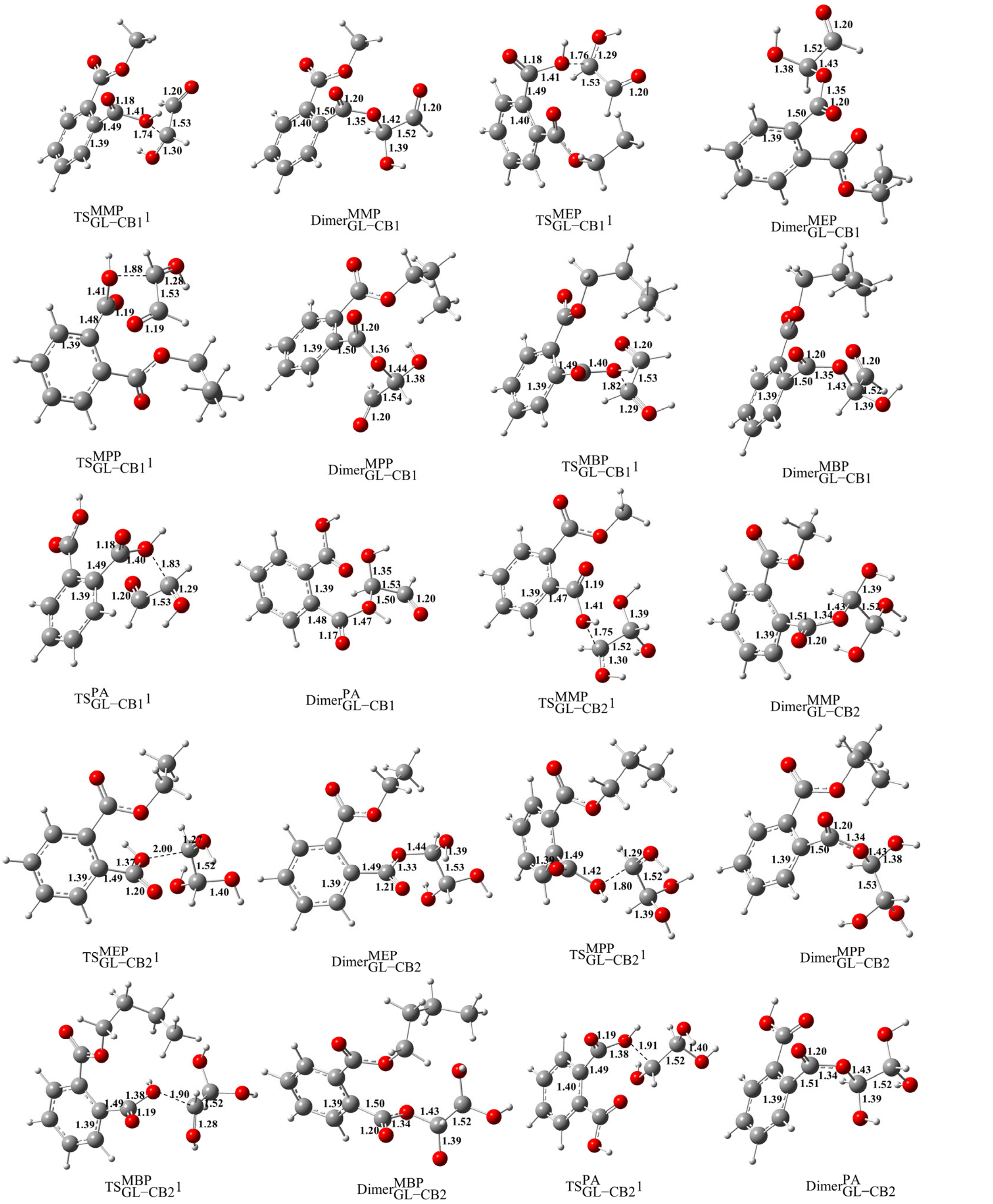

Figure S14: The geometries of the TSs and ester-like dimer for the oligomerization reaction between GL-CBs (denoted as GL-CB1 and GL-CB2) and five hydrolysis products (MMP, MEP, MPP, MBP, and PA) obtained at the level of the M06-2X/6-311G(d,p). The number is bond length (in Å). C; O; H

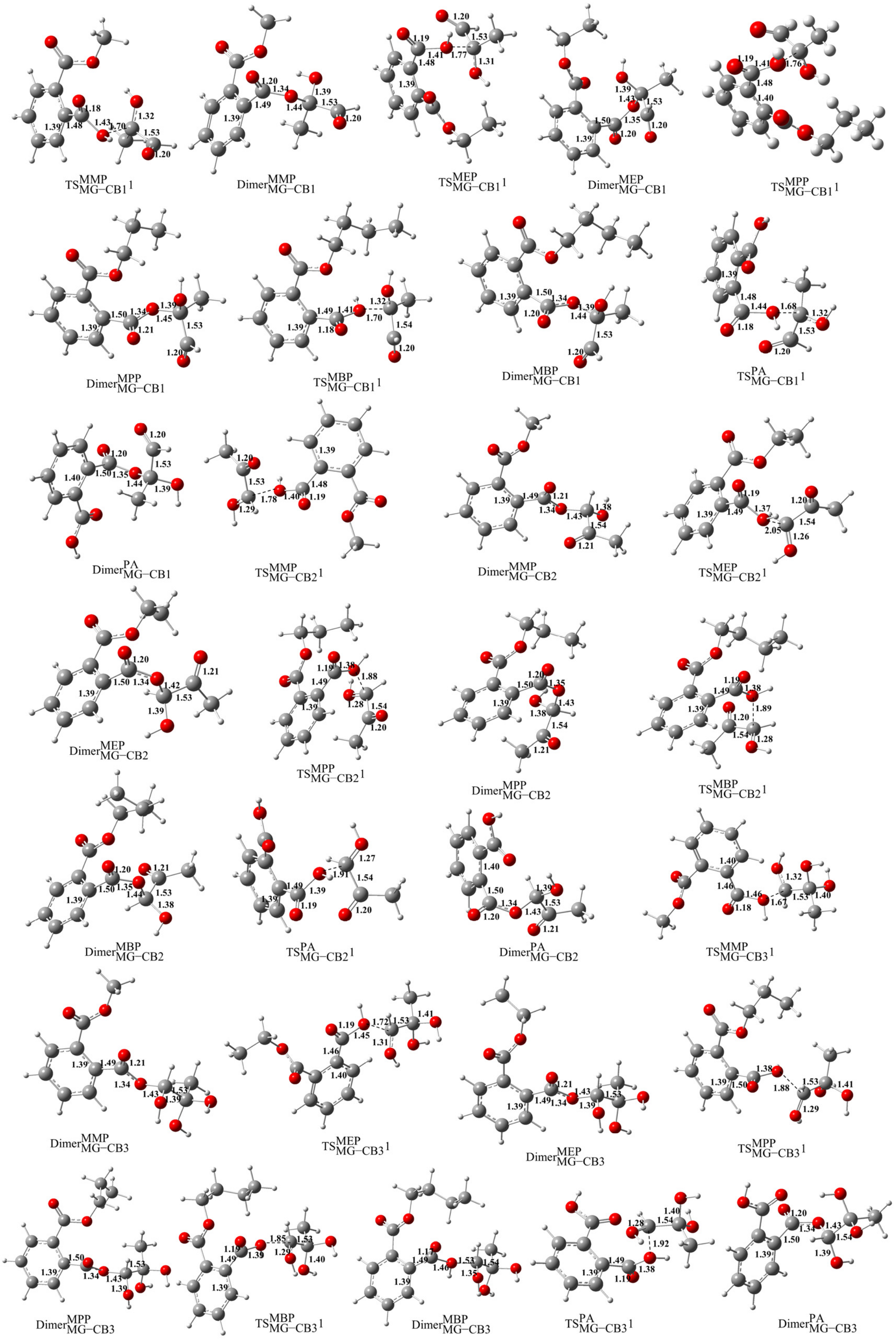

Figure S15: The geometries of the TSs and ester-like dimer for the oligomerization reaction between MG-CBs (denoted as MG-CB$_1$, MG-CB$_2$, and GL-CB$_3$) and five hydrolysis products (MMP, MEP, MPP, MBP, and PA) obtained at the level of the M06-2X/6-311G(d,p). The number is bond length (in Å). C; O; H

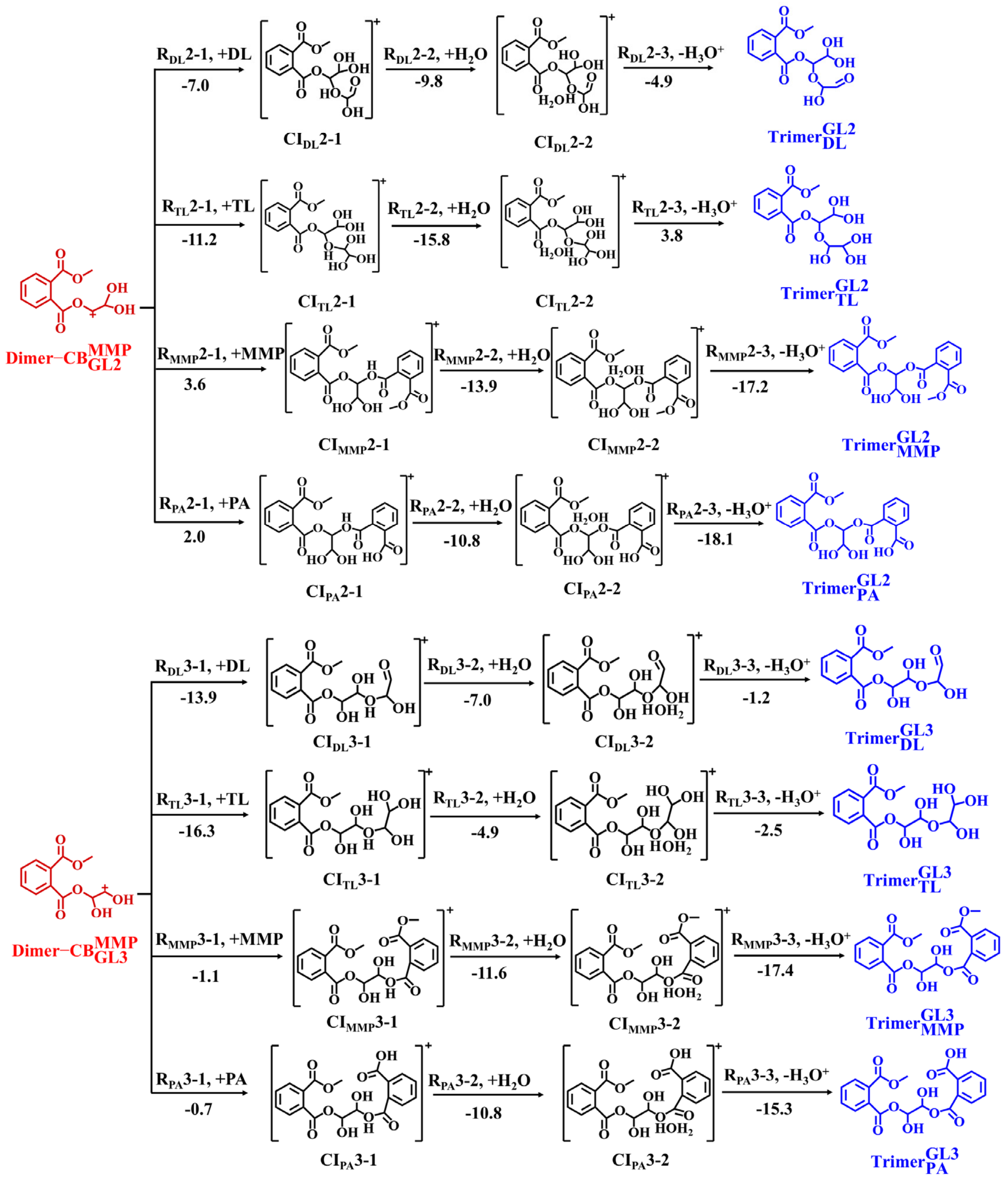

Figure S16: The subsequent association reactions of Dimer-CB $^{MMP}_{GL\text{-}CB2}$ and Dimer-CB $^{MMP}_{GL\text{-}CB3}$. The number is the ΔG_r in each reaction (in kcal mol^{-1})

Table S1. The k values for the hydrolysis reactions of DMP, DEP, DPP and DBP at 298 K

k	DMP	DEP	DPP	DBP
R_{OH^-}-1-2[a]	2.59×10^{3}	2.35×10^{3}	6.04×10^{3}	5.49×10^{3}
R_{H^+}1-5[a]	1.46×10^{-13}	2.32×10^{-13}	1.69×10^{-13}	2.12×10^{-14}
R_{H2O}1[b]	2.58×10^{-28}	1.31×10^{-28}	1.40×10^{-27}	3.05×10^{-28}
R_{OH^-}-1-1[b]	8.19×10^{3}	8.19×10^{3}	1.07×10^{3}	4.59×10^{2}
R_{H^+}1-1[b]	1.09×10^{9}	1.06×10^{9}	1.04×10^{9}	1.04×10^{9}
R_{H^+}1-2[b]	1.72×10^{9}	1.71×10^{9}	1.71×10^{9}	1.67×10^{9}
R_{H^+}1-3[b]	1.60×10^{9}	1.55×10^{9}	1.53×10^{9}	1.54×10^{9}
R_{H^+}2-1[b]	1.09×10^{9}	1.07×10^{9}	1.05×10^{9}	1.04×10^{9}
R_{H^+}2-2[b]	1.46×10^{0}	3.18×10^{-1}	3.51×10^{-2}	2.96×10^{-2}
R_{H^+}2-3[b]	1.85×10^{9}	1.80×10^{9}	1.80×10^{9}	1.76×10^{9}

[a]The unit of the rate constant for unimolecular reaction is s^{-1}. [b]The unit of the rate constant for bimolecular reaction is $M^{-1}\ s^{-1}$.

Table S2. The $t_{1/2}$ values at pH = 6 for the hydrolysis reactions of DMP, DEP, DPP and DBP at 298 K

$t_{1/2}$ (s)	DMP	DEP	DPP	DBP
R_{OH^-}1-2[a]	2.68×10^{-4}	2.95×10^{-4}	1.15×10^{-4}	1.26×10^{-4}
R_{H^+}1-5[a]	4.75×10^{12}	2.99×10^{12}	4.10×10^{12}	3.27×10^{13}
R_{H2O}1[b]	1.98×10^{37}	3.40×10^{37}	2.83×10^{36}	1.17×10^{37}
R_{OH^-}1-1[b]	8.46×10^{3}	8.46×10^{3}	6.48×10^{4}	1.51×10^{5}
R_{H^+}1-1[b]	6.36×10^{-4}	6.54×10^{-4}	6.66×10^{-4}	6.66×10^{-4}
R_{H^+}1-2[b]	4.03×10^{-4}	4.05×10^{-4}	4.05×10^{-4}	4.15×10^{-4}
R_{H^+}1-3[b]	4.33×10^{-4}	4.47×10^{-4}	4.53×10^{-4}	4.50×10^{-4}
R_{H^+}2-1[b]	6.36×10^{-4}	6.48×10^{-4}	6.60×10^{-4}	6.66×10^{-4}
R_{H^+}2-2[b]	4.75×10^{5}	2.18×10^{6}	1.97×10^{7}	2.34×10^{7}
R_{H^+}2-3[b]	3.75×10^{-4}	3.85×10^{-4}	3.85×10^{-4}	3.94×10^{-4}

Table S3: The *k* values for the association reactions between GL-CBs (s=1, 2) and MMP, MEP, MPP, MBP, and PA at 298 K.

Reactions	Pathways	$\Delta G^{\ddagger}$ (kcal mol^{-1})	k (M^{-1} s^{-1})
$GL\text{-}CB_1 + MMP \rightarrow CI^{MMP}_{GL\text{-}CB1}1$	$d\text{-}R^{MMP}_{GL\text{-}CB1}1$	10.1	3.39×10^{8}
$CI^{MMP}_{GL\text{-}CB1}1 + H_2O \rightarrow CI^{MMP}_{GL\text{-}CB1}2$	$d\text{-}R^{MMP}_{GL\text{-}CB1}2$		2.77×10^{9}
$GL\text{-}CB_1 + MEP \rightarrow CI^{MEP}_{GL\text{-}CB1}1$	$d\text{-}R^{MEP}_{GL\text{-}CB1}1$	8.9	1.12×10^{9}
$CI^{MEP}_{GL\text{-}CB1}1 + H_2O \rightarrow CI^{MMP}_{GL\text{-}CB1}2$	$d\text{-}R^{MEP}_{GL\text{-}CB1}2$		1.84×10^{9}
$GL\text{-}CB_1 + MPP \rightarrow CI^{MPP}_{GL\text{-}CB1}1$	$d\text{-}R^{MPP}_{GL\text{-}CB1}1$	15.4	5.28×10^{4}
$CI^{MPP}_{GL\text{-}CB1}1 + H_2O \rightarrow CI^{MPP}_{GL\text{-}CB1}2$	$d\text{-}R^{MPP}_{GL\text{-}CB1}2$		1.77×10^{9}
$GL\text{-}CB_1 + MBP \rightarrow CI^{MBP}_{GL\text{-}CB1}1$	$d\text{-}R^{MBP}_{GL\text{-}CB1}1$	10.0	3.87×10^{8}
$CI^{MBP}_{GL\text{-}CB1}1 + H_2O \rightarrow CI^{MBP}_{GL\text{-}CB1}2$	$d\text{-}R^{MBP}_{GL\text{-}CB1}2$		1.83×10^{9}
$GL\text{-}CB_1 + PA \rightarrow CI^{PA}_{GL\text{-}CB1}1$	$d\text{-}R^{PA}_{GL\text{-}CB1}1$	10.4	2.22×10^{8}
$CI^{PA}_{GL\text{-}CB1}1 + H_2O \rightarrow CI^{PA}_{GL\text{-}CB1}2$	$d\text{-}R^{PA}_{GL\text{-}CB1}2$		1.95×10^{9}
$GL\text{-}CB_2 + MMP \rightarrow CI^{MMP}_{GL\text{-}CB2}1$	$d\text{-}R^{MMP}_{GL\text{-}CB2}1$	11.7	2.75×10^{7}
$CI^{MMP}_{GL\text{-}CB2}1 + H_2O \rightarrow CI^{MMP}_{GL\text{-}CB2}2$	$d\text{-}R^{MMP}_{GL\text{-}CB2}2$		1.87×10^{9}
$GL\text{-}CB_2 + MEP \rightarrow CI^{MEP}_{GL\text{-}CB2}1$	$d\text{-}R^{MEP}_{GL\text{-}CB2}1$	11.0	8.73×10^{7}
$CI^{MEP}_{GL\text{-}CB2}1 + H_2O \rightarrow CI^{MEP}_{GL\text{-}CB2}2$	$d\text{-}R^{MEP}_{GL\text{-}CB2}2$		1.79×10^{9}
$GL\text{-}CB_2 + MPP \rightarrow CI^{MPP}_{GL\text{-}CB2}1$	$d\text{-}R^{MPP}_{GL\text{-}CB2}1$	13.1	2.60×10^{6}
$CI^{MPP}_{GL\text{-}CB2}1 + H_2O \rightarrow CI^{MPP}_{GL\text{-}CB2}2$	$d\text{-}R^{MPP}_{GL\text{-}CB2}2$		1.83×10^{9}
$GL\text{-}CB_2 + MBP \rightarrow CI^{MBP}_{GL\text{-}CB2}1$	$d\text{-}R^{MBP}_{GL\text{-}CB2}1$	12.9	3.65×10^{6}
$CI^{MBP}_{GL\text{-}CB2}1 + H_2O \rightarrow CI^{MBP}_{GL\text{-}CB2}2$	$d\text{-}R^{MBP}_{GL\text{-}CB2}1$		1.94×10^{9}
$GL\text{-}CB_2 + PA \rightarrow CI^{PA}_{GL\text{-}CB2}1$	$d\text{-}R^{PA}_{GL\text{-}CB1}1$	11.0	8.72×10^{7}
$CI^{PA}_{GL\text{-}CB2}1 + H_2O \rightarrow CI^{PA}_{GL\text{-}CB2}2$	$d\text{-}R^{PA}_{GL\text{-}CB2}2$		1.85×10^{9}

Table S4: The *k* values for the association reactions between MG-CBs (s=1, 2, 3) and MMP, MEP, MPP, MBP, and PA at 298 K.

Reactions	Pathways	$\Delta G^{\ddagger}$ (kcal mol^{-1})	k (M^{-1} s^{-1})
$\text{MG-CB}_1 + \text{MMP} \rightarrow \text{CI}^{\text{MMP}}_{\text{MG-CB1}}1$	$\text{d-R}^{\text{MMP}}_{\text{MG-CB1}}1$	18.6	2.33×10^{2}
$\text{CI}^{\text{MMP}}_{\text{MG-CB1}}1 + \text{H}_2\text{O} \rightarrow \text{CI}^{\text{MMP}}_{\text{MG-CB1}}2$	$\text{d-R}^{\text{MMP}}_{\text{MG-CB1}}2$		1.87×10^{9}
$\text{MG-CB}_1 + \text{MEP} \rightarrow \text{CI}^{\text{MEP}}_{\text{MG-CB1}}1$	$\text{d-R}^{\text{MEP}}_{\text{MG-CB1}}1$	17.4	1.78×10^{3}
$\text{CI}^{\text{MEP}}_{\text{MG-CB1}}1 + \text{H}_2\text{O} \rightarrow \text{CI}^{\text{MEP}}_{\text{MG-CB1}}2$	$\text{d-R}^{\text{MEP}}_{\text{MG-CB1}}2$		1.81×10^{9}
$\text{MG-CB}_1 + \text{MPP} \rightarrow \text{CI}^{\text{MEP}}_{\text{MG-CB1}}1$	$\text{d-R}^{\text{MEP}}_{\text{MG-CB1}}1$	18.4	3.26×10^{2}
$\text{CI}^{\text{MPP}}_{\text{MG-CB1}}1 + \text{H}_2\text{O} \rightarrow \text{CI}^{\text{MPP}}_{\text{MG-CB1}}2$	$\text{d-R}^{\text{MPP}}_{\text{MG-CB1}}2$		1.95×10^{9}
$\text{MG-CB}_1 + \text{MBP} \rightarrow \text{CI}^{\text{MBP}}_{\text{MG-CB1}}1$	$\text{d-R}^{\text{MBP}}_{\text{MG-CB1}}1$	18.0	6.44×10^{2}
$\text{CI}^{\text{MBP}}_{\text{MG-CB1}}1 + \text{H}_2\text{O} \rightarrow \text{CI}^{\text{MBP}}_{\text{MG-CB1}}2$	$\text{d-R}^{\text{MBP}}_{\text{MG-CB1}}2$		1.88×10^{9}
$\text{MG-CB}_1 + \text{PA} \rightarrow \text{CI}^{\text{PA}}_{\text{MG-CB1}}1$	$\text{d-R}^{\text{PA}}_{\text{MG-CB1}}1$	17.8	9.04×10^{2}
$\text{CI}^{\text{PA}}_{\text{MG-CB1}}1 + \text{H}_2\text{O} \rightarrow \text{CI}^{\text{PA}}_{\text{MG-CB1}}2$	$\text{d-R}^{\text{PA}}_{\text{MG-CB1}}2$		2.17×10^{9}
$\text{MG-CB}_2 + \text{MMP} \rightarrow \text{CI}^{\text{MMP}}_{\text{MG-CB2}}1$	$\text{d-R}^{\text{MMP}}_{\text{MG-CB2}}1$	11.0	8.69×10^{7}
$\text{CI}^{\text{MMP}}_{\text{MG-CB2}}1 + \text{H}_2\text{O} \rightarrow \text{CI}^{\text{MMP}}_{\text{MG-CB2}}2$	$\text{d-R}^{\text{MMP}}_{\text{MG-CB2}}2$		1.96×10^{9}
$\text{MG-CB}_2 + \text{MEP} \rightarrow \text{CI}^{\text{MEP}}_{\text{MG-CB2}}1$	$\text{d-R}^{\text{MEP}}_{\text{MG-CB2}}1$	11.3	5.36×10^{7}
$\text{CI}^{\text{MEP}}_{\text{MG-CB2}}1 + \text{H}_2\text{O} \rightarrow \text{CI}^{\text{MEP}}_{\text{MG-CB2}}2$	$\text{d-R}^{\text{MEP}}_{\text{MG-CB2}}2$		1.98×10^{9}
$\text{MG-CB}_2 + \text{MPP} \rightarrow \text{CI}^{\text{MEP}}_{\text{MG-CB2}}1$	$\text{d-R}^{\text{MEP}}_{\text{MG-CB2}}1$	11.4	4.53×10^{7}
$\text{CI}^{\text{MPP}}_{\text{MG-CB2}}1 + \text{H}_2\text{O} \rightarrow \text{CI}^{\text{MPP}}_{\text{MG-CB2}}2$	$\text{d-R}^{\text{MPP}}_{\text{MG-CB2}}2$		1.77×10^{9}
$\text{MG-CB}_2 + \text{MBP} \rightarrow \text{CI}^{\text{MBP}}_{\text{MG-CB2}}1$	$\text{d-R}^{\text{MBP}}_{\text{MG-CB2}}1$	9.7	5.58×10^{8}
$\text{CI}^{\text{MBP}}_{\text{MG-CB2}}1 + \text{H}_2\text{O} \rightarrow \text{CI}^{\text{MBP}}_{\text{MG-CB2}}2$	$\text{d-R}^{\text{MBP}}_{\text{MG-CB2}}2$		1.78×10^{9}
$\text{MG-CB}_2 + \text{PA} \rightarrow \text{CI}^{\text{PA}}_{\text{MG-CB2}}1$	$\text{d-R}^{\text{PA}}_{\text{MG-CB2}}1$	10.5	1.91×10^{8}
$\text{CI}^{\text{PA}}_{\text{MG-CB2}}1 + \text{H}_2\text{O} \rightarrow \text{CI}^{\text{PA}}_{\text{MG-CB2}}2$	$\text{d-R}^{\text{PA}}_{\text{MG-CB2}}2$		1.91×10^{9}
$\text{MG-CB}_3 + \text{MMP} \rightarrow \text{CI}^{\text{MMP}}_{\text{MG-CB3}}1$	$\text{d-R}^{\text{MMP}}_{\text{MG-CB3}}1$	17.5	1.50×10^{3}
$\text{CI}^{\text{MMP}}_{\text{MG-CB3}}1 + \text{H}_2\text{O} \rightarrow \text{CI}^{\text{MMP}}_{\text{MG-CB3}}2$	$\text{d-R}^{\text{MMP}}_{\text{MG-CB3}}2$		3.13×10^{9}
$\text{MG-CB3} + \text{MEP} \rightarrow \text{CI}^{\text{MEP}}_{\text{MG-CB3}}1$	$\text{d-R}^{\text{MEP}}_{\text{MG-CB3}}1$	17.3	2.11×10^{3}
$\text{CI}^{\text{MEP}}_{\text{MG-CB3}}1 + \text{H}_2\text{O} \rightarrow \text{CI}^{\text{MEP}}_{\text{MG-CB3}}2$	$\text{d-R}^{\text{MEP}}_{\text{MG-CB3}}2$		3.12×10^{9}
$\text{MG-CB}_3 + \text{MPP} \rightarrow \text{CI}^{\text{MPP}}_{\text{MG-CB3}}1$	$\text{d-R}^{\text{MPP}}_{\text{MG-CB3}}1$	15.4	5.28×10^{4}
$\text{CI}^{\text{MPP}}_{\text{MG-CB3}}1 + \text{H}_2\text{O} \rightarrow \text{CI}^{\text{MPP}}_{\text{MG-CB3}}2$	$\text{d-R}^{\text{MPP}}_{\text{MG-CB3}}2$		2.01×10^{9}
$\text{MG-CB}_3 + \text{MBP} \rightarrow \text{CI}^{\text{MBP}}_{\text{MG-CB3}}1$	$\text{d-R}^{\text{MBP}}_{\text{MG-CB3}}1$	14.7	1.73×10^{5}
$\text{CI}^{\text{MBP}}_{\text{MG-CB3}}1 + \text{H}_2\text{O} \rightarrow \text{CI}^{\text{MBP}}_{\text{MG-CB3}}2$	$\text{d-R}^{\text{MBP}}_{\text{MG-CB3}}2$		2.56×10^{9}
$\text{MG-CB}_3 + \text{PA} \rightarrow \text{CI}^{\text{PA}}_{\text{MG-CB3}}1$	$\text{d-R}^{\text{PA}}_{\text{MG-CB3}}1$	14.3	3.41×10^{5}
$\text{CI}^{\text{PA}}_{\text{MG-CB3}}1 + \text{H}_2\text{O} \rightarrow \text{CI}^{\text{PA}}_{\text{MG-CB3}}2$	$\text{d-R}^{\text{PA}}_{\text{MG-CB3}}2$		2.78×10^{9}

References

1. Eyring, H. The activated complex in chemical reactions. *J. Chem. Phys.* **1935**, *3*, 107-115.
2. Galano, A.; Alvarez-Idaboy, J.R. Guanosine plus OH Radical Reaction in Aqueous Solution: A Reinterpretation of the UV-vis Data Based on Thermodynamic and Kinetic Calculations. *Org. Lett.* **2009**, *11*, 5114-5117.
3. Okuno, Y. Theoretical investigation of the mechanism of the baeyer-villiger reaction in nonpolar solvents. *Chem.-Eur. J.* **1997**, *3*, 212-218.
4. Collins, F.C.; Kimball, G.E. Diffusion-controlled reaction rates. *J. Colloid Sci.* **1949**, *4*, 425-437.
5. Einstein, A. Über die von der molekularkinetischen Theorie der Wärme geforderte Bewegung von in ruhenden Flüssigkeiten suspendierten Teilchen. *Ann. Phys.* **1905**, *322*, 549-560.
6. Jiang, B.; Zhao, S.Z.; Chen, W.; Tian, L.L.; Hu, W.W.; Li, J.; Zhang, G. Intrinsic Chemical Drivers of Organic Aerosol Volatility: From Experimental Insights to Model Predictions. *J. Geophys. Res.-Atmos.* **2024**, *129*, 13.
